# Alleviation of Gut Inflammation by Cdx2/Pxr Pathway in a Mouse Model of Chemical Colitis

**DOI:** 10.1371/journal.pone.0036075

**Published:** 2012-07-16

**Authors:** Wei Dou, Subhajit Mukherjee, Hao Li, Madhukumar Venkatesh, Hongwei Wang, Sandhya Kortagere, Ariel Peleg, Sridhar S. Chilimuri, Zheng-Tao Wang, Ying Feng, Eric R. Fearon, Sridhar Mani

**Affiliations:** 1 Departments of Medicine and Genetics, Albert Einstein College of Medicine, New York, New York, United States of America; 2 Shanghai Key Laboratory of Formulated Chinese Medicines and MOE Key Laboratory for Standardization Chinese Medicine, Institute of Chinese Materia Medica, Shanghai University of TCM, Shanghai, China; 3 Department of Microbiology and Immunology, Drexel University College of Medicine, Philadelphia, Pennsylvania, United States of America; 4 Department of Medicine, Bronx Lebanon Hospital Center, Bronx, New York, United States of America; 5 Institute of Chinese Materia Medica, Shanghai University of TCM, Shanghai, China; 6 Division of Molecular Medicine and Genetics, Department of Internal Medicine, University of Michigan Medical School, Ann Arbor, Michigan; 7 Division of Molecular Medicine and Genetics, Department of Internal Medicine, Human Genetics and Pathology, University of Michigan Medical School, Ann Arbor, Michigan, United States of America; Ohio State University, United States of America

## Abstract

Pregnane X Receptor (PXR), a master regulator of drug metabolism and inflammation, is abundantly expressed in the gastrointestinal tract. Baicalein and its *O*-glucuronide baicalin are potent anti-inflammatory and anti-cancer herbal flavonoids that undergo a complex cycle of interconversion in the liver and gut. We sought to investigate the role these flavonoids play in inhibiting gut inflammation by an axis involving PXR and other potential factors. The consequences of PXR regulation and activation by the herbal flavonoids, baicalein and baicalin were evaluated *in vitro* in human colon carcinoma cells and *in vivo* using wild-type, *Pxr-*null, and humanized (*hPXR*) PXR mice. Baicalein, but not its glucuronidated metabolite baicalin, activates PXR in a Cdx2-dependent manner *in vitro,* in human colon carcinoma LS174T cells, and in the murine colon *in vivo*. While both flavonoids abrogate dextran sodium sulfate (DSS)-mediated colon inflammation *in vivo*, oral delivery of a potent bacterial β-glucuronidase inhibitor eliminates baicalin’s effect on gastrointestinal inflammation by preventing the microbial conversion of baicalin to baicalien. Finally, reduction of gastrointestinal inflammation requires the binding of Cdx2 to a specific proximal site on the PXR promoter. Pharmacological targeting of intestinal PXR using natural metabolically labile ligands could serve as effective and potent therapeutics for gut inflammation that avert systemic drug interactions.

## Introduction

For centuries, the roots of *Scutellaria baicalensis* Georgi (Labiatae) have been used for the treatment of allergic, inflammatory and cancer-related diseases in China and Japan [1], [2]. In these roots, the principal ingredients, in terms of both abundance and effectiveness as an anti-oxidant, are baicalein (5,6,7-trihydroxyflavone) and its 7-glucuronic acid conjugate, baicalin [2]. Both of these flavonoids have shown remarkable *in vitro* and *in vivo* pharmacodynamic activities related to their anti-oxidant, anti-inflammatory, anti-viral and anti-bacterial properties [2]. These constituents have a striking propensity to nonspecifically bind to proteins, thus masking their “true” pharmacodynamic targets [3]. Among several molecular targets described for baicalein/baicalin (e.g., transcription induction, enzyme inhibition/induction, reactive oxygen species) [4], [5], very few of these targets have emerged as important pharmacodynamic targets [6], [7]. Indeed, these properties of flavones have stimulated analog discovery efforts to optimize pharmacodynamic properties [3], [8].

Flavonoids, baicalin and baicalein abrogate inflammation in various organs, [9] as well as in inflammatory bowel disease (IBD) [10]. However, the role for baicalin in inflammatory bowel disease remains controversial [11]. Since, no mechanism for this effect has been reported, our efforts focused on whether the adopted orphan nuclear receptor, Pregnane X Receptor (PXR) [12], [13] could potentially mediate flavonoid effects in the gut. This receptor is abundantly expressed in the intestine and liver of mammals and rodents [14]. It is a master regulator of xenobiotic detoxification, and more recently, it has been shown to regulate (abrogate) inappropriate gut inflammation [15]. Furthermore, unlike any known receptor system described to date, PXR has the largest and most promiscuous ligand-binding pocket [16]. Indeed, ligand binding and the activation of PXR can be stereo (enantiomer)-specific with respect to the parent compound and metabolites [17], [18].

Using human colon cancer-derived cells, we found that baicalein, in contrast to baicalin, induces PXR through Cdx2. Baicalein also activated PXR in PXR-transactivation assays. Using animal models, we demonstrated that both baicalein and baicalin abrogate intestinal inflammation and reduce TNFα and IL-6 mRNA abundance. However, the beneficial effect of baicalein on intestinal inflammation was observed in wild-type (*Pxr* ^+/+^) and *hPXR* mice but not in *Pxr-*null (*Pxr* ^−/−^) mice. Baicalin, also abrogated inflammation, but this effect was abolished with oral gavage of Inh1 (Inhibitor 1), a novel microbe specific β-glucuronidase inhibitor [19] that prevents baicalin from converting to baicalein [20]. Further, we demonstrate that Cdx2 binds and activates RNA Polymerase (Pol) II recruitment on a specific site in the proximal PXR promoter (∼1.4 kb from the start site). In summary, we found that baicalein, but not its major metabolite (glucuronide) baicalin, protects from inappropriate intestinal inflammation *in vivo* through the induction of Cdx2 and PXR expression.

## Results

### Baicalein, in Contrast to Baicalin, Induces PXR and Cdx2 in Colon Cancer Cells

**Figure 1:**
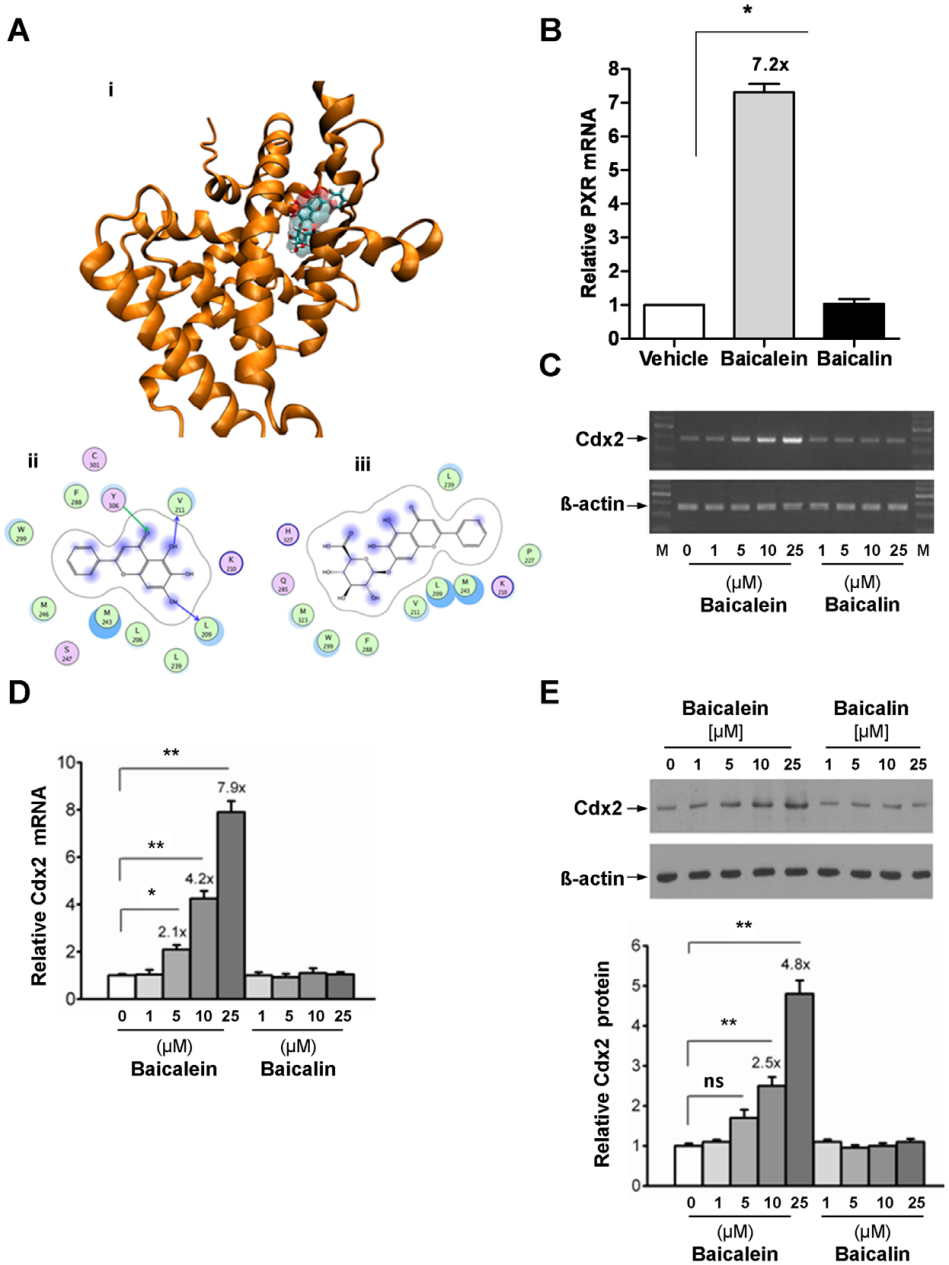
Baicalein, in contrast to baicalin, docks within the PXR ligand binding domain and induces PXR and Cdx2 in LS174T colon carcinoma cells. (A) Molecular docking of baicalein and baicalin to PXR ligand binding domain (LBD). Figure (i) represent molecular docking of baicalein to PXR LBD. The 2D schematic diagrams shown in (ii, baicalein) and (iii, baicalin) were generated using the LIGX module of MOE program. The binding site residues are colored by their nature, with hydrophobic residues in green, polar residues in purple and charged residues highlighted with bold contours. Blue spheres and contours indicate matching regions between ligand and receptors. Hydrogen bond interactions are shown by green and blue arrows for side chain and main chain interactions, respectively. (B) LS174T cells were exposed to 0.1% DMSO (vehicle) and baicalein (25 µM) or baicalin (25 µM) for 48 hours and total RNA was isolated for PXR mRNA expression analysis by real-time quantitative (RT-qPCR). (C & D) LS174T cells were exposed to different concentrations of baicalein or baicalin as illustrated, for 48 hours and total RNA isolated for Cdx2 mRNA expression analysis by (C) semi-quantitative polymerase chain reaction and by (D) RT-qPCR. β-actin was used as internal control. (E, top panel) Representative western blot of Cdx2 from the same experiment as in (C). (E, bottom panel) Absolute band intensity was quantified for each lanes of the western blot as in figure (E, top panel), using Image J software. Histogram, mean ± SEM. *P<.01; ** *P*<.001; ns, not significant.

Although baicalein is known to activate PXR [21], the extent to which baicalin and baicalein affect PXR transcription and function is unknown. Molecular modeling of baicalein and baicalin showed that baicalein is predicted to have greater ligand binding residue interactions via H-bonds, which are absent for baicalin (Figure 1A). Notably, PXR transactivation studies in DPX2 cells showed that baicalein activates PXR, while baicalin had no PXR transactivation potential (Figure S1A) and minimal cellular toxicity (Figure S1B). Some herbal drug extracts (e.g., protocatechuic aldehyde) have been shown to activate and induce PXR [22]. Thus, we tested whether baicalein and its glucuronide metabolite, baicalin would induce PXR expression in LS174T colon cancer cells *in vitro*. Baicalein induced PXR (7.2 fold over control) while baicalin had no effect on PXR mRNA abundance (Figure 1B). Similar data were shown for another comparable Cdx2 expressing colon cell line, LoVo (Figure S1C).

Since flavonoids are known to regulate transcription factor (TF) activity directly (e.g., β-catenin/Tcf) [23] or indirectly (e.g., baicalein inhibition of NFκB) [24], and that PXR mRNA abundance is regulated at the level of transcription by intestine-specific and non-specific TFs [25], we chose to focus on the mechanism by which flavonoids regulate PXR transcription. PXR and the intestine specific homeobox protein caudal type homeobox 2 (Cdx2) are co-expressed in enterocytes [26], [27], with Cdx2 as a central regulator of intestinal differentiation [28], [29]. Furthermore, Cdx2 acts as a key regulator of intestinal homeostasis [30], [31], similar to our observations in *Pxr* ^−/−^ mice *in vivo* (unpublished observations). Given these possible similarities shared by Cdx2 and PXR, we sought to determine whether Cdx2 could transactivate PXR in 293T cells. We found that PXR promoter activity increased with Cdx2 dosage (Figure S1D). Baicalein was found to increase the expression of Cdx2 mRNA in LS174T cells (Figure 1C & D). A similar trend was also observed for Cdx2 protein expression (Figure 1E), albeit the expression abundance was lower than that observed for Cdx2 mRNA (4.8-fold versus 7.9-fold, respectively) (Figure 1E, bottom panel). Baicalin had no effect on Cdx2 mRNA or protein expression (Figure 1C–E). To further corroborate the apparent link between Cdx2 and PXR, several intestinal cells (HCT 116, SW 948, SW 403 and LoVo) expressing varying amounts of Cdx2 protein, were exposed to flavonoids. In contrast to baicalin, baicalein induced PXR expression in cell lines expressing Cdx2 protein (Figure S1C).

### Baicalein, in Contrast to Baicalin, Induces PXR mRNA Expression through Cdx2

**Figure 2:**
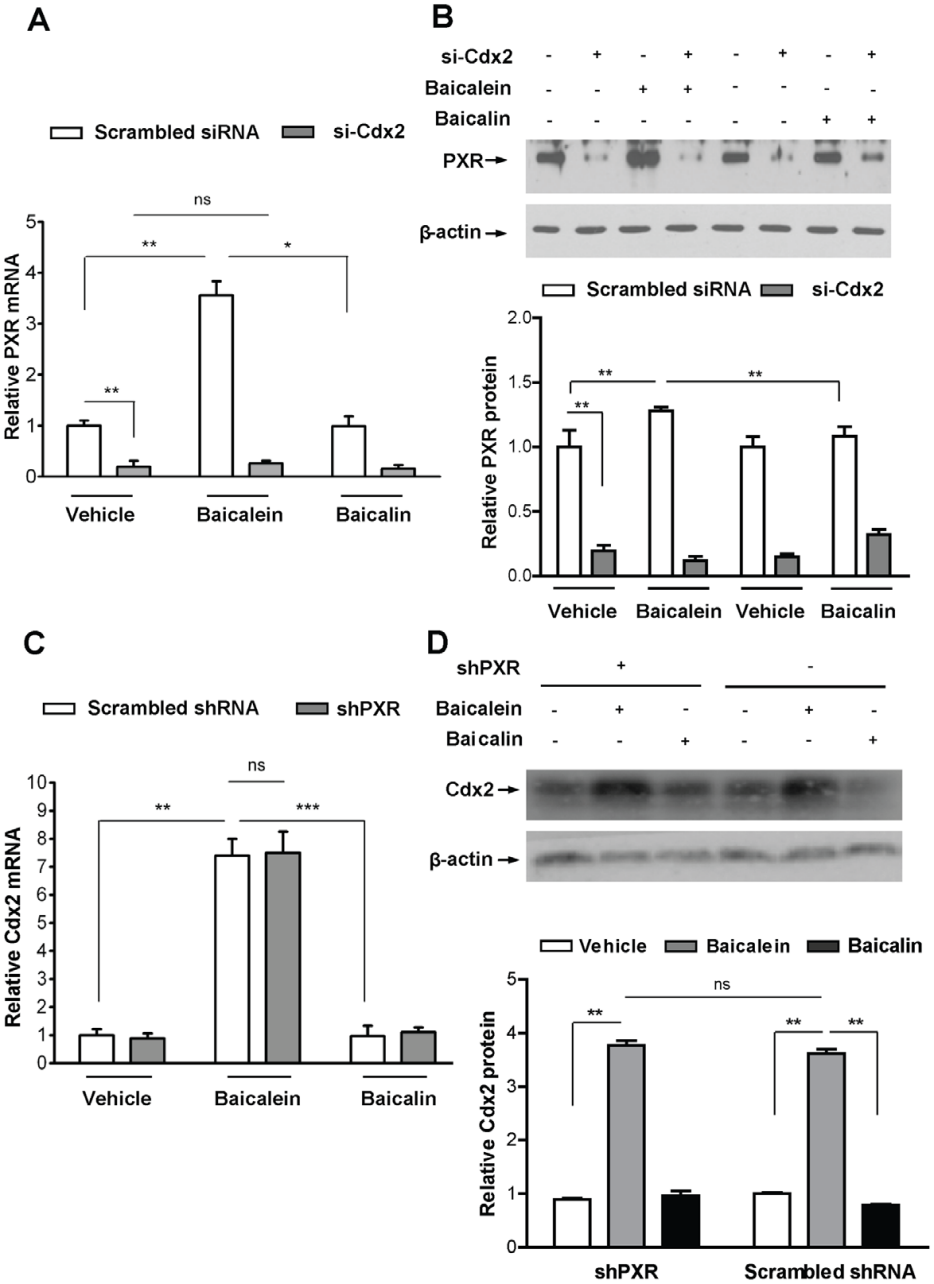
Baicalein, in contrast to baicalin, induces PXR and Cdx2 mRNA. (A) Scrambled or Cdx2 siRNA (si-Cdx2) transfected LS174T colon cancer cells were exposed to 0.1% DMSO (vehicle), baicalein (25 µM) or baicalin (25 µM) for 48 hours. mRNA levels of PXR were quantified by RT-qPCR. (B, top panel) Representative western blot of PXR from the same experiment as in (A). (B, bottom panel) Absolute band intensity was quantified for each lanes of the western blot as in figure (B, top panel), using Image J software. (C) Scrambled or PXR shRNA (shPXR) transduced LS174T cells, were exposed to 0.1% DMSO (vehicle), baicalein (25 µM) or baicalin (25 µM) for 48 hours. Cdx2 mRNA levels were quantified by RT-qPCR. (D, top panel) Representative western blot of Cdx2 from the same experiment as in (C). (D, bottom panel) Absolute band intensity was quantified for each lanes of the western blot as in figure (D, top panel), using Image J software. Histogram, mean ± SEM. *P<.05; ** *P*<.01;*** *P*<.001; ns, not significant.

Next, we examined whether Cdx2 is necessary for baicalein-mediated induction of PXR in LS174T cells. Cdx2 silencing in LS174T cells (Figure S2) significantly reduced PXR mRNA (Figure 2A) and protein (Figure 2B) abundance. Baicalein induced PXR mRNA (Figure 2A) and protein (Figure 2B) in scrambled siRNA transfected but not in Cdx2 silenced LS174T cells. To further validate the relationship between Cdx2 function and PXR gene expression, HT-29-derived lines with tightly regulated Cdx2 activity [32], were used to confirm that PXR is a direct target of Cdx2 (Figure S3). As a corollary, DLD-1 derived lines with constitutively expressing Cdx2 shRNA were used to confirm reciprocal effects of Cdx2 and PXR (Figure S4). Conjointly, these results strongly argue for a direct transcriptional effect of Cdx2 on PXR transcription.

### Baicalein, in Contrast to Baicalin, Induces Cdx2 Independent of PXR Expression

Next, we examined whether PXR is necessary for flavonoid-mediated induction of Cdx2. We found that PXR knockdown in LS174T cells (Figure S5) had no effect on the basal Cdx2 mRNA (Figure 2C), protein abundance (Figure 2D), and baicalein-mediated induction of Cdx2 mRNA (Figure 2C) or its protein abundance (Figure 2D). Similarly, PXR knockdown did not alter the effect of baicalin on Cdx2 mRNA expression levels (Figure 2C) nor its protein abundance (Figure 2D).

### The Flavones Baicalein and Baicalin Abrogate Intestinal Inflammation in a Mouse Model of Colitis through a PXR-dependent Mechanism

**Figure 3:**
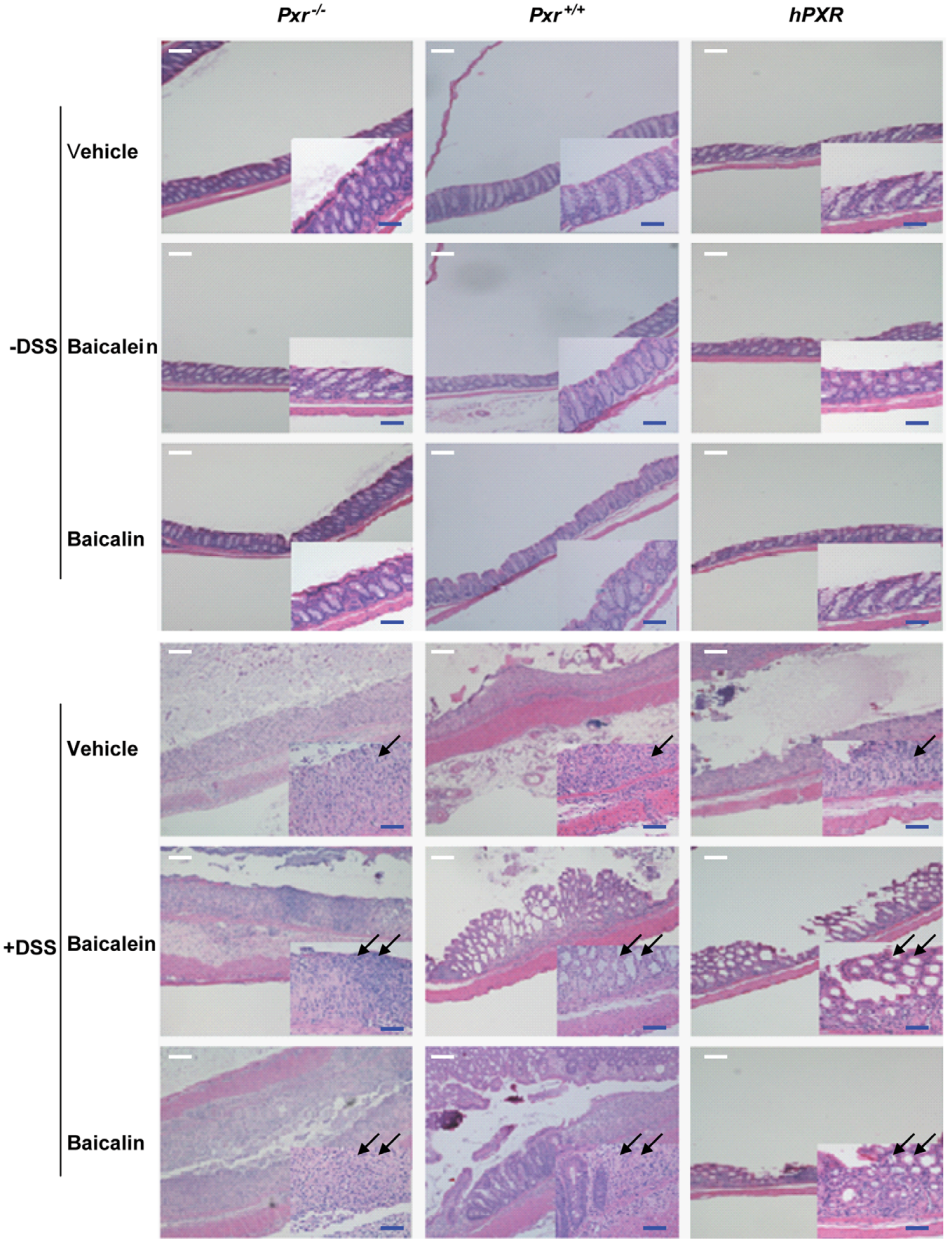
Protective effects of flavonoids in DSS-induced colitis in mice. Histopathological examination of the colon of mice exposed to DSS and/or flavones (Baicalein, Baicalin) is illustrated. The histology (inset shows higher magnification views) represents the prototypical injury to the colon induced by DSS. *Pxr^+/+^*: wild-type mice; *Pxr^−/−^*: *Pxr*-null mice; *hPXR*: humanized PXR mice. –DSS: DSS untreated mice; +DSS: DSS treated mice; single black arrow: vehicle exposed mucosa; double black arrow: flavonoids exposed mucosa. Scale bar, 50 µM.

**Figure 4:**
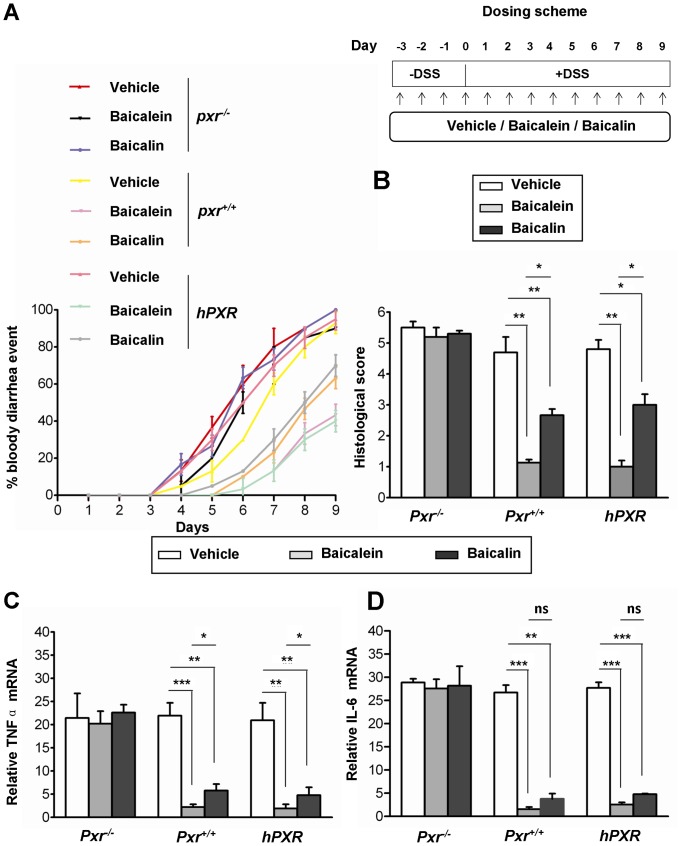
Flavonoids protect against DSS-induced bloody diarrhea, colitis score and inflammatory cytokine expressions. (A) Mice exposed to DSS, as represented in Figure 3 according to the dosing scheme illustrated in upper right panel of (A), were evaluated for the presence of bloody diarrhea after flavonoids treatment. Data plotted as percentage (%) of total mice that had bloody diarrhea on different days of DSS treatment. (B) The histologic score was determined on day 9, when mice were sacrificed. (C & D) Total RNA was isolated from colons of DSS treated mice as in figure (A & B) and analyzed for (C) TNFα and (D) IL-6 mRNA expressions by RT-qPCR. Histogram and data points, mean ± SEM. *P<.05; **P<.01; ***P<.001; ns, not significant.

The dextran sodium sulfate (DSS) model of murine colitis closely simulates the human condition and is one commonly used model of human IBD. Baicalein has been previously shown to abrogate inflammation in such a model [10]. Similarly, PXR ligands also abrogate inflammation [15]. We investigated whether the anti-inflammatory actions of baicalein were dependent on *Pxr in vivo*. DSS exposed mice were treated with vehicle or flavonoids (baicalein, baicalin). The histopathology of the colon was examined by hematoxylin/eosin, as shown in Figure 3. The insets illustrate the effect of DSS (vehicle treated) on the intestinal mucosa where it induces significant inflammatory infiltrates (black arrow; Figure 3), regardless of the presence of *Pxr*. Indeed, both baicalein and baicalin protected against DSS-mediated intestinal inflammation and crypt loss; however, this effect was lost in the absence of *Pxr* (double black arrow; Figure 3). The humanized mice showed effects and responses similar to those of wild-type mice (*Pxr^+/+^*). Clinically, DSS-exposed mice treated with baicalein and baicalin, showed a reduced cumulative incidence of “bloody diarrhea” as compared to the vehicle-exposed mice, that was more pronounced in *Pxr* ^+/+^ and *hPXR* as compared to the *Pxr* ^−/−^ mice (Figure 4A). Using the same mice on day 9, the colon histological (inflammation) score was determined. Baicalein significantly reduced the histological score in DSS-exposed *Pxr^+/+^* and *hPXR* mice but not in *Pxr* ^−/−^ mice (Figure 4B). Surprisingly, baicalin significantly reduced the histological score in DSS-exposed *Pxr^+/+^* and *hPXR* mice but not in *Pxr* ^−/−^ mice (Figure 4B). However, the effects of baicalein were significantly greater than those of baicalin (Figure 4B). Messenger RNA expression levels of the mediators of inflammation, TNFα and IL-6, were also assessed in the intestinal mucosa of the same mice. Baicalein and baicalin significantly reduced both TNFα (Figure 4C) and IL-6 (Figure 4D) mRNA expression levels in DSS-treated *Pxr^+/+^* and *hPXR* mice but not in *Pxr* ^−/−^ mice. Once again, the effects of baicalein were significantly greater than those of baicalin (Figure 4D).

### The Flavones Baicalein and Baicalin Induce Cdx2 in the Intestinal Mucosa through a PXR-Independent Mechanism *in vivo*

**Figure 5:**
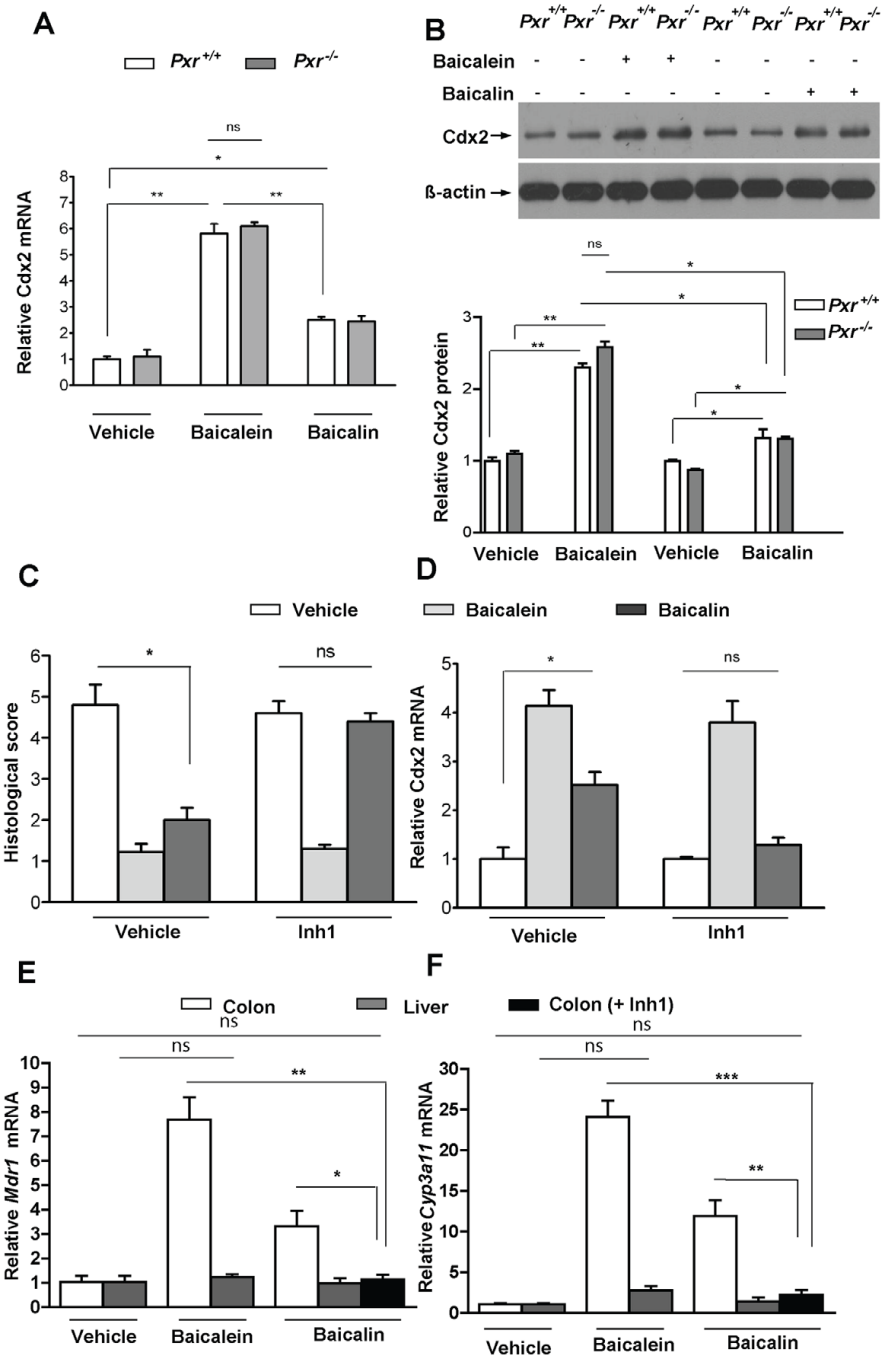
Flavonoids induce Cdx2 mRNA and protein regardless of *Pxr* genotype in murine intestinal mucosa. (A & B) *Pxr* ^+/+^ and *Pxr* ^−/−^ mice (DSS untreated) exposed to flavones (baicalein, baicalin), as represented in Figure 3, were evaluated for (A) Cdx2 mRNA expression by RT-qPCR and (B, top panel) Cdx2 protein abundance by western blot. (B, bottom panel) Absolute band intensity was quantified for each lanes of the western blot as in figure (B, top panel), using Image J software. (C & D) *Pxr* ^+/+^ mice (DSS treated) pre-exposed to Inh1 and subsequently treated with flavones (baicalein, baicalin), as in Figure 3, were evaluated for (C) histological score and (D) Cdx2 mRNA expression (analyzed by RT-qPCR) in colon isolated on day 9 of DSS treatment. (E & F) *Pxr* ^+/+^ mice were treated with flavonoids (baicalein, baicalin) and mRNA expressions of *Mdr1* and *Cyp3a11* were analyzed in colon and liver. Flavonoids (baicalein, baicalin) have no effect on induction of (E) *Mdr1* and (F) *Cyp3a11* mRNA in *Pxr* ^+/+^ mice liver; however, they robustly induce gene expression in the colon. Inh1 abolishes baicalin’s effect on induction of *Mdr1* and *Cyp3a11* mRNA in colon, as assessed by RT-qPCR. Histogram, mean ± SEM. * *P*<.05; ** *P*<.01; ns, not significant.

Both baicalein and baicalin induced Cdx2 mRNA expression (Figure 5A) and protein abundance (Figure 5B), which was independent of *Pxr* content in the intestinal mucosa (Figure 5B, bottom panel). Moreover, the effects of baicalein were significantly greater than those of baicalin (Figure 5A & B).

### Baicalin’s Effect on Intestinal Inflammation and Cdx2 Expression is Mediated through the Formation of Baicalein *in vivo*

The *in vivo* data suggested that baicalin, which is not an efficient inducer or activator of PXR, was surprisingly dependent on PXR for its anti-inflammatory actions in the gut. However, baicalin, is converted to baicalein by microbial β-glucuronidase *in vivo* [2]. Baicalin is a good substrate for microbial β-glucuronidase (K_m_ ∼0.038–0.01), and it is paradoxically a weak inhibitor of the enzyme (Brenda, www. Brenda-enzymes.org) [33]. To test whether baicalin inhibited microbial β-glucuronidase *in vivo*, protein (enzyme source) was extracted from mouse fecal pellets and incubated with 7-Ethyl-10-hydroxycamptothecin glucuronide (SN-38-G) (substrate) at various time intervals [19]. The resulting product, SN-38 (aglycone), was measured by spectrophotometry (Emission wavelength ∼425 nm) (Figure S6A). β-glucuronidase activity was minimally inhibited by baicalin in murine fecal extracts. However, a recently described inhibitor of microbial β-glucuronidase, Inh1 [19], was significantly more efficient at inhibiting β-glucuronidase (Figure S6B). To determine whether the anti-inflammatory actions of baicalin were indeed due to its *in* *situ* conversion to baicalein, mice were treated with Inh1, before being treated with baicalin. Inh1 dramatically normalized the baicalin-mediated reduction in histological scores in DSS-exposed mice (Figure 5C) and Cdx2 mRNA expression in untreated mice (Figure 5D).

### Flavone Concentrations that Inhibit Intestinal Inflammation *in vivo* do not Activate PXR in the Liver

The flavonoids baicalein and baicalin are thought to exert systemic drug interaction effects through enzyme induction/inhibition [2]. To determine whether baicalein and baicalin administered at a dose and schedule of 20 mg/kg daily for 13 days led to the systemic activation of PXR, non-DSS exposed mice were exposed to vehicle or flavonoids. The colon and liver were excised after 13 days, and total RNA was extracted for real-time quantitative PCR of the classical PXR target genes, *Mdr1* and *Cyp3a11*. Both baicalein and baicalin induced *Mdr1* (Figure 5E) and *Cyp3a11* (Figure 5F) mRNA expression in the colon but not in the liver. Given that there was significant conversion of baicalin to baicalein in the gut (*Mdr1* and *Cyp3a11* mRNA were not induced in the colon when Inh1 was present, Figure 5E & F, respectively) and that this conversion was responsible for the *in vivo* effects of baicalin on intestinal inflammation and Cdx2 expression, we conclude that the effect of baicalin on *Mdr1* and *Cyp3a11* in the colon was due to its conversion to baicalein.

### Cdx2 Binds to Specific PXR Proximal Promoter Element and Recruits Pol II

**Figure 6:**
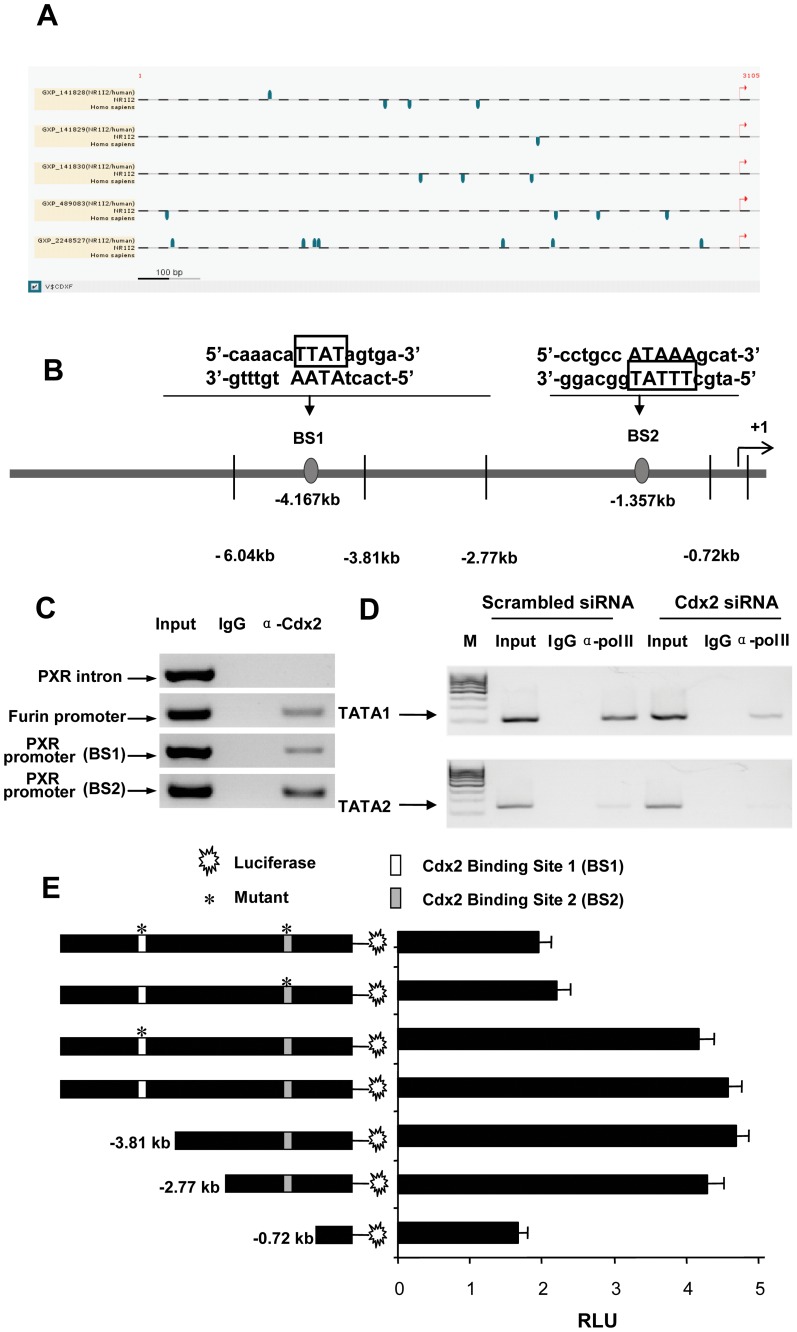
Cdx2 binds to two specific endogenous binding sites on the proximal PXR promoter. (A) Schematic details of the sites (blue marker) that were identified with a high match [matrix scores (≥0.85)], represent potential functional Cdx2 binding sites on PXR promoter. (Note: three potential Cdx1 binding sites, which are also identified on analysis, were excluded from consideration). The *in silico* analysis identified a total of 19 potential Cdx1/2 binding sites, of which, we verified endogenous binding for two sites, BS1 and BS2 by (C) Chromatin Immunoprecipitation (ChIP) assays. BS, Cdx2 binding site. ChIP assays were performed with Cdx2 and non-specific IgG antibodies and Cdx2 binding was assessed on PXR promoter (BS1 and BS2), PXR intron (negative control) and Furin promoter (positive control) regions. (D) Pol II ChIP assays were performed, using chromatin extracted from LS174T cells that were exposed to scrambled or Cdx2 siRNA (si-Cdx2). Pol II occupancy was assessed on PXR proximal promoter (TATA box) region, using Pol II and non-specific IgG antibodies as a control. (E) Cdx2 transactivation assay was performed in 293T cells, transfected with ∼6 kb PXR promoter reporter constructs (with or without deletion and mutations, as shown in the figure) and Cdx2 expression plasmid. Data expressed as RLU. RLU, relative light unit. Histogram, mean ± SD.

A bioinformatics analysis revealed several putative Cdx2 binding sites on the 3 kb proximal promoter of the PXR (-) strand (Figure 6A). A ChIP analysis revealed that only sites 1 and 2 (denoted BS1 and BS2 in Figure 6B, respectively) bind to endogenous Cdx2 (Figure 6C). The EMSA results show that both BS1 (Figure S7) and BS2 (Figure S8) bind Cdx2. The EMSA showed several non-specific bands, which have been sequentially excluded as they were not specific to Cdx2 (Figure S7 and S8).

To determine whether Cdx2 is a functional component of PXR transcription (i.e., whether it binds to the PXR promoter but does not initiate transcription through Pol II recruitment), Pol II ChIP assays were performed using nuclear extracts from LS174T cells that were silenced for Cdx2 expression. The results showed that Pol II was efficiently recruited to the PXR proximal promoter (TATA box) in scrambled siRNA transfected cells; however, qualitatively Pol II was reduced in cells in which Cdx2 had been silenced (Figure 6D). To further validate the functional consequence of Cdx2 binding to BS1 and BS2 of the ∼6 kb PXR promoter, we constructed several PXR deletion mutants and performed PXR transactivation assays with the Cdx2 expression plasmid in 293T cells. The ∼6 kb promoter activity was nearly identical to the ∼2.8 kb deletion construct; however, the ∼0.7 kb promoter had a clearly reduced (by ∼60%) transactivation potential compared to the ∼2.8 kb promoter, suggesting that BS2 is likely the functionally active binding site for Cdx2 on the PXR promoter (Figure 6E). To validate this finding, we serially mutated BS1 and/or BS2. The BS1 mutation had no effect on PXR promoter activity in the presence of Cdx2; however, mutation of BS2 and/or BS1 plus BS2 significantly reduced the ∼6.0 kb PXR promoter activity (by ∼60%), which was identical to that observed with the ∼0.8 kb deletion construct (Figure 6E).

## Discussion

Using genetic mouse models of drug metabolism and inflammation, we identified PXR as a critical target of baicalein in abrogating IBD. Of note, PXR has been previously implicated as an important target for gut inflammation [34], [35], [36]. Moreover, PXR haplotypes have been implicated in childhood IBD [34]. Certain drugs used in the clinic, such as rifaximin and prednisone, either activate or induce PXR expression and are associated with a beneficial outcome in intestinal immune homeostasis [37], [38]. Baicalein/baicalin has been previously shown to abrogate intestinal inflammation in a mouse model of colitis, although the mechanisms were not established [10], [11]. In this context, we have shown that baicalein activates human and likely, mouse PXR, albeit the latter has been inferred through *in vivo* studies on gut inflammation only. We confirm that Cdx2, a known intestinal cell differentiation factor [39], is induced by baicalein. Additionally, Cdx2 directly induces PXR promoter activity and mRNA expression in intestinal cells. Altogether, we provide compelling evidence that baicalein exerts its effects on inflammation through a Cdx2/PXR pathway.

We also showed that baicalin, a glucuronide-modified analogue of baicalein, is unable to induce Cdx2 and/or PXR and that it does not activate PXR *in vitro* or *in vivo*. The apparent anti-inflammatory effects of oral baicalin in mice are largely due to its conversion to the aglycone, baicalein, through the actions of microbial β-glucuronidases [40]. Indeed, even the effects of oral baicalin are modest compared with baicalein. Furthermore, oral baicalein at doses sufficient to suppress intestinal inflammation does not induce PXR target genes in the liver. Thus, we propose that oral baicalein could achieve concentrations in intestinal cells that could sufficiently abrogate inflammation locally. However, upon systemic absorption, baicalein is completely metabolized and excreted as its glucuronide or sulfate conjugate. We also demonstrate that baicalein is unable to induce PXR target genes in the liver, which is consistent with data showing that baicalin is unable to either induce or activate PXR. These data suggest that baicalein will not induce systemic drug interactions through PXR at the doses used to abrogate intestinal inflammation in mice. The clinical and translational relevance of our results are consistently supported by published data, demonstrating that baicalein is significantly metabolized in both humans and rodents. The glucuronide metabolites are major metabolites in both species [2], [41], [42].

We demonstrated that Cdx2 binds to a single element on the proximal promoter of PXR. Our data show that a careful analysis of specific sites may reveal important and functional gene regulatory elements. These data could be used to further determine the molecular mechanisms governing Cdx2-mediated PXR transduction. However, Cdx2 may function independently as a suppressor of inflammation, perhaps through non-transcriptional mechanisms [30], [43], and/or induce multiple morphogenetic changes that counter-balance the effects of mucosal inflammation [31]. These possibilities warrant further mechanistic studies. Finally, it is to be noted that PXR inhibits NF-*κ*B activity [44]. Mutual repression between steroid and xenobiotic receptor and NF-*κ*B signaling pathways links xenobiotic metabolism and inflammation, which could play an important independent role in abrogating intestinal inflammation and perhaps in conjunction with the newer observations implicating Cdx2. In this context, we have not assessed the impact of baicalein on activation of other nuclear receptors (e.g., liver X receptor or LXR, farnesoid X receptor or FXR), which independently affect intestinal inflammatory pathways. Farnesoid X receptor activation inhibits inflammation and preserves the intestinal barrier in inflammatory bowel disease [45]. Probiotics modulate intestinal expression of nuclear receptor and provide counter-regulatory signals to inflammation-driven adipose tissue activation [46].

On the basis of our findings, we propose that knowledge of baicalein’s *in vivo* clinical pharmacology could aid its use in patients with inflammatory bowel disease. However, the effects of baicalein on drug absorption as a result of nontargeted effects could still be operational [47]. Accordingly, it would be feasible and warrant the synthesis of potent PXR-activating baicalein analogs while still maintaining or even gaining metabolic lability (i.e., instability due to presence of increased sites for glucuronidation) [8]. Conversely, re-formulations of baicalein for colonic delivery and release may also be feasible [48].

## Materials and Methods

The cell lines were procured and cultured according to the guidelines of American Type Culture Collection (ATCC, Rockville, MD, USA) or as per the information provided with the respective cell line**. Cell lines, and Reagents,** ***In Silico*** **Binding Site Analysis, Molecular Docking, Gene Reporter Assay** and **PXR knock-down assay** details are mentioned in File S1.

### Transient Transfection Assay

293T cells were seeded at 70% confluence into 96-well plates. After an overnight incubation, transient transfection was performed using Lipofectamine 2000 reagent according to the manufacturer’s recommendation and as described in File S1. Details of cell viability assay, DPX2 based PXR transactivation and Cdx2 siRNA transfection assays are described in File S1.

### Chromatin Immunoprecipitation (ChIP) Assay

We used a fast ChIP method as previously published [49]. For complete procedure, see File S1. Details of the PCR conditions and primer sequences are described in Table S1.

### Electrophoretic Mobility Shift Assay (EMSA)

The gel shift assays were performed as previously described [26]. For complete procedure, see File S1. Probe sequences for EMSA are described in Table S2.

### PXR Promoter Deletion Constructs

Three 6.04-kb deletion constructs (−3.81 kb, −2.77 kb and −0.72 kb) containing different lengths of the 5′-flanking sequences of the PXR gene were prepared for the luciferase transcription assay of the human PXR promoter and Site-Directed Mutagenesis were carried out as described in File S1.

### PXR Mouse Models

*Pxr*-null (*Pxr^−/−^*) mice (8–10 weeks of age) and humanized (*hPXR*) PXR mice (mice that express the human PXR gene in a *Pxr*-null background) were generously provided by Dr. Jeff Staudinger (University of Kansas, Lawrence, KS) and Dr. Wen Xie (University of Pittsburgh, Pittsburgh, PA), respectively [50], [51]. Wild-type controls of the same age, gender and strain (C57BL/6) were purchased from Jackson Laboratory (Bar Harbor, Maine). In a subset of experiments, both *Pxr*-null and *hPXR* mice were backcrossed to wild-type controls, and after at least 5 generations, wild-type litters were obtained to serve as controls in our experiments. Animal experiments were carried out following Institutional Animal Care and Use guidelines approved by the Institutional Animal Care and Use Committee (IACUC) of the Albert Einstein College of Medicine, New York.

### DSS Mouse Model of Colitis

Mice were fed with 4% (wt/vol) DSS (MW 36–50 KDa, MP Biomedical LLC, Solon, OH) dissolved in sterile, distilled water (vehicle control) ad libitum for the duration of the experiment (days 0–9). Flavonoids were administered to the animals (n = 8–15/genotype/treatment group) by oral gavage (20 mg/kg/day in distilled water, respectively) for 3 days prior to the DSS treatment; the flavonoid administration continued until the end of the DSS treatment (days −3–9), as described previously [10]. For the β-glucuronidase inhibitor study, mice (n = 10/treatment group) were exposed to Inh1 (10 µg twice a day) on day −3 by oral gavage [19].

### Clinical and Histological Scores of Colitis

Clinical signs of colitis, such as rectal bleeding, diarrhea, bloody stool and histological score were assessed and reported as a scored event as described previously [19].

### RNA Isolation and Real-Time Quantitative (q) PCR

Total RNA was isolated using previously published methods [52], and RT-qPCR was carried out as described in File S1. Gene expression changes were calculated using the comparative C_t_ method with β-actin as the reference gene and vehicle treated cells (or indicated as control) as the calibrator.

### Semi-quantitative, Immunoprecipitation, Western Blot Analysis, β-Glucuronidase Enzyme Assay and Immunohistochemistry

For complete procedure, see File S1.

### Statistical Analysis

Statistical analyses were performed by unpaired Student’s *t*-test. For comparison of more than two groups of data, a 1-way ANOVA was performed. *P* values less than.05 were considered to be statistically significant (GraphPad *Prism version* 4).

## Supporting Information

Figure S1 **Baicalein, but not baicalin, activates and induces PXR** ***in vitro*** **.** (A) The DPX2 assay (see Materials and Methods) was used to assess PXR transactivation and (B) cell viability. (C) Individual colon cancer cells lines with varying abundance of Cdx2 protein were used to determine the effect of flavonoids (baicalein, baicalin) on induction of PXR mRNA. Bottom panel shows expression of Cdx2 protein in these cell lines. (D) PXR transactivation assay in 293T cells co-expressing Cdx2 and −3.81 kb PXR reporter. Histogram and data points, mean ± SEM.
(PDF)

Figure S2 **Cdx2 knockdown in LS174T colon cancer cells.** (A) LS174T cells co-transfected with GFP expressing plasmid and scrambled siRNA or si-Cdx2 with images captured under appropriate filters (Phase contrast and GFP). The middle panel shows fold Cdx2 mRNA expression by RT-qPCR. β-actin was used as internal control. Gene expression changes were calculated using the comparative Ct method with β-actin as the reference gene and scrambled siRNA transfected cells as the calibrator. The bottom panel shows a western blot of Cdx2 from nuclear extract from the same cells. Histogram, mean ± SEM. Scale bar, 100 µm; ** *P*<0.02
(PDF)

Figure S3 **Cdx2 directly targets and induces the PXR promoter.** (A) Schematic diagram illustrating possible mechanisms by which Cdx2 induces PXR mRNA, through its (1) direct action or (2, 3) indirect action(s) on the PXR promoter. BS: Cdx2 binding site on the PXR promoter. (B) Western blot of cell lysates from two clones (c1, c2) of HT-29/Cdx2-ER cells (obtained after sequential passage) with (+) or without (-) exposure to 4-OHT (hydroxytamoxifen) for Cdx2 and β-actin. The right panel shows time-dependent induction of PXR mRNA as assessed by RT-qPCR. (C) Immunoprecipitation blot of PXR from HT-29/Cdx2-ER cells exposed to cycloheximide and/or 4-OHT. Right panel shows fold expression of PXR mRNA by RT-qPCR. β-actin was used as internal or loading control for western blots. Gene expression changes were calculated using the comparative Ct method with β-actin as the reference gene and vehicle treated cells (or indicated as 0) as the calibrator. Histogram, mean ± SD.
(PDF)

Figure S4 **Cdx2 directly targets and induces the PXR promoter.** (A) Left panel shows western blot of Cdx2 in cell lysates prepared from DLD-1 pGIPZ(c) (control cells) and shCdx2 infected cells. Right panel shows fold mRNA expression of Cdx2 and PXR by RT-qPCR from the same experiment. (B) Immunoprecipitation blot of PXR from control DLD-1 pGIPZ(c) and shCdx2 infected cells exposed to cycloheximide and/or 4-OHT. Right panel shows fold expression of PXR mRNA by RT-qPCR. β-actin was used as internal or loading control for western blots. Gene expression changes were calculated using the comparative Ct method with β-actin as the reference gene and vehicle treated cells (or indicated as 0) as the calibrator. Histogram, mean ± SD.
(PDF)

Figure S5 **PXR knockdown in LS174T colon cancer cells.** (A) LS174T cells co-transduced with GFP expressing plasmid and scrambled shRNA or shPXR with images captured under appropriate filters (Phase contrast and GFP). The middle panel shows fold PXR mRNA expression by RT-qPCR. β-actin was used as internal control. Gene expression changes were calculated using the comparative Ct method with β-actin as the reference gene and scrambled shRNA transfected cells as the calibrator. The bottom panel shows a western blot for PXR from nuclear extract from the same cells. Histogram, mean ± SEM. Scale bar, 100 µm; ** *P*<0.02
(PDF)

Figure S6 **Baicalin does not decrease SN-38 glucuronide (SN-38G) emission spectra upon incubation with mouse feces.** (A) SN-38G emission spectra in HEPES buffer. (B) Co-incubation of mouse feces with vehicle (HEPES buffer), Inh1 (10 µM), or Baicalin (10 µM).
(PDF)

Figure S7 **Cdx2 binds to binding Site1 (BS1) oligonucleotide probe by electrophoretic mobility shift assay (EMSA).** (A) Black arrows demonstrate bands upon incubation of respective probes with and without LS174T colon cancer cell line nuclear extract. The lower arrow indicates band common to the positive control and PXR probe lane. (B) Black arrow (bold) demonstrates loss of band upon addition of 50-fold excess of cold PXR probe. (C) Black arrow (bold) demonstrates supershift band, identical to that observed in positive control lane, with the addition of Cdx2 antibody. (D) Black arrow (bold) demonstrates loss of band upon incubation of LS174T nuclear extracts with BS1 mutant oligo. -, negative control oligo; +, positive control oligo; wt, wild-type or PXR probe sequence from PXR promoter containing BS1; mt, PXR probe sequence mutant of BS1.
(PDF)

Figure S8 **Cdx2 binds to binding Site2 (BS2) oligonucleotide probe by electrophoretic mobility shift assay (EMSA).** (A) Black arrows demonstrate bands upon incubation of respective probes with and without LS174T colon cancer cell line nuclear extract. The lower arrow indicates band common to the positive control and PXR probe lane. (B) Black arrow (bold) demonstrates loss of band upon addition of 50-fold excess of cold PXR probe. (C) Black arrow (bold) demonstrates supershift band, identical to that observed in positive control lane, with the addition of anti-Cdx2 antibody. (D) Black arrow (bold) demonstrates loss of band upon incubation of LS174T nuclear extracts with BS2 mutant oligo. -, negative control oligo; +, positive control oligo; wt, wild-type or PXR probe sequence from PXR promoter containing BS2; mt, PXR probe sequence mutant of BS2.
(PDF)

Table S1 Primer sequences for ChIP assay.
(DOC)

Table S2 Probe Sequences for EMSA.
(DOC)

File S1 File contains the following: Supporting Information Methods including primer sequences and shRNA sequences, Supporting Information Results and Supporting Information References.
(DOC)
