# Supplementary figures and images for: Alleviation of Gut Inflammation by Cdx2/Pxr Pathway in a Mouse Model of Chemical Colitis
Source: PLoS One. 2012 Jul 16;7(7):e36075. doi: 10.1371/journal.pone.0036075 (PMC3398007; doi:10.1371/journal.pone.0036075)

**A**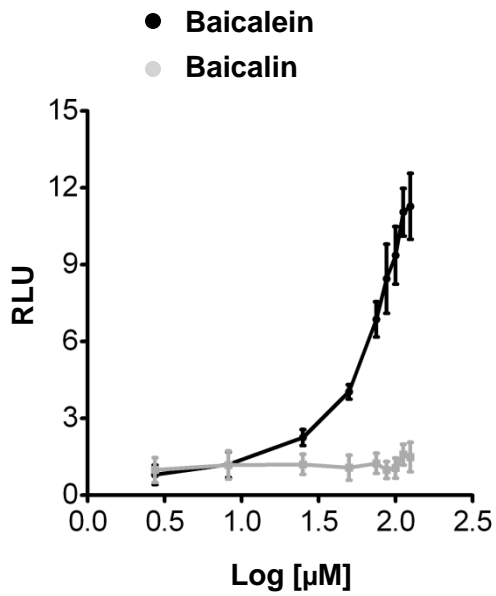**B**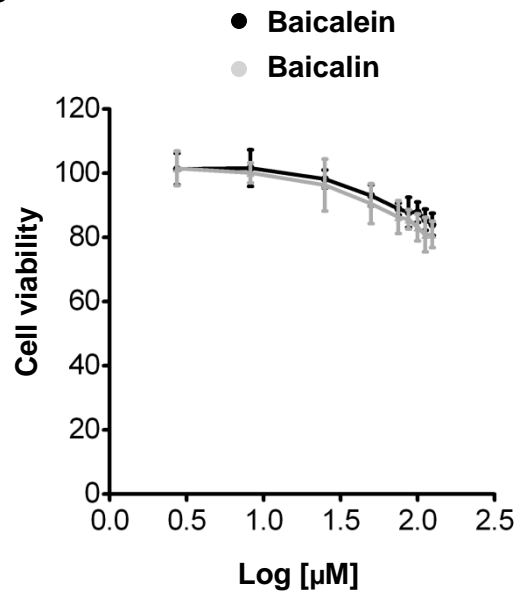**C**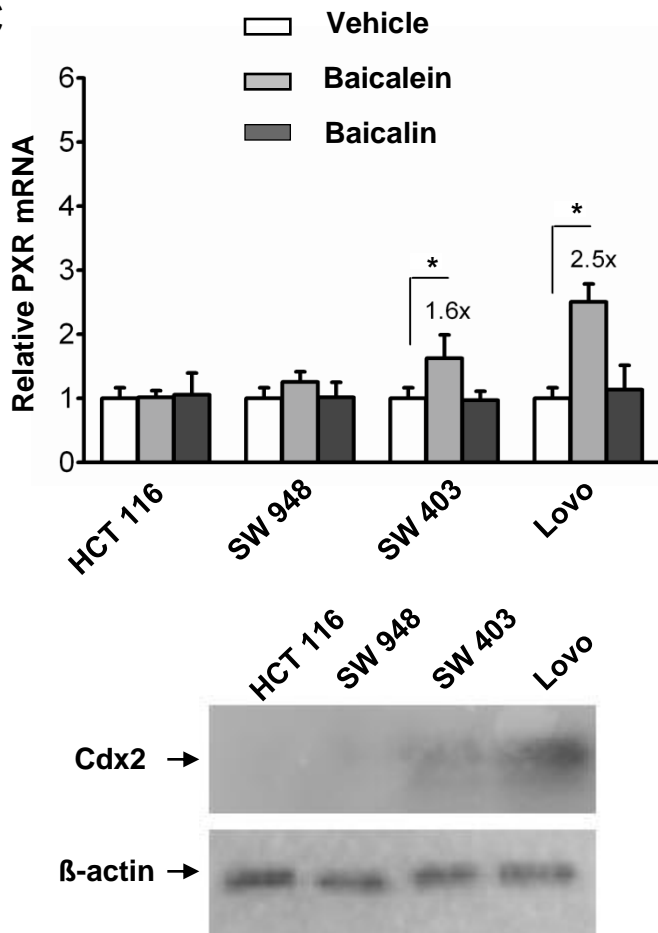**D**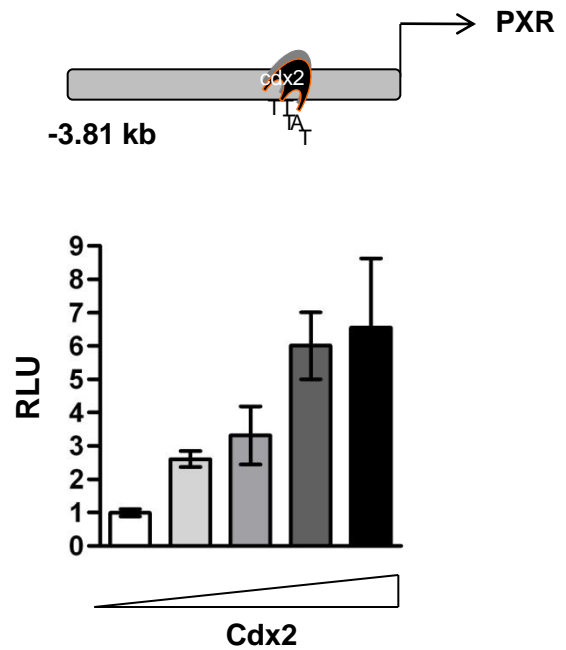**Figure S1**

Supplement: Figure S1 — Baicalein, but not baicalin, activates and induces PXR in vitro . (A) The DPX2 assay (see Materials and Methods) was used to assess PXR transactivation and (B) cell viability. (C) Individual colon cancer cells lines with varying abundance of Cdx2 protein were used to determine the effect of flavonoids (baicalein, baicalin) on induction of PXR mRNA. Bottom panel shows expression of Cdx2 protein in these cell lines. (D) PXR transactivation assay in 293T cells co-expressing Cdx2 and −3.81 kb PXR reporter. Histogram and data points, mean ± SEM. (PDF) [file pone.0036075.s001.pdf]

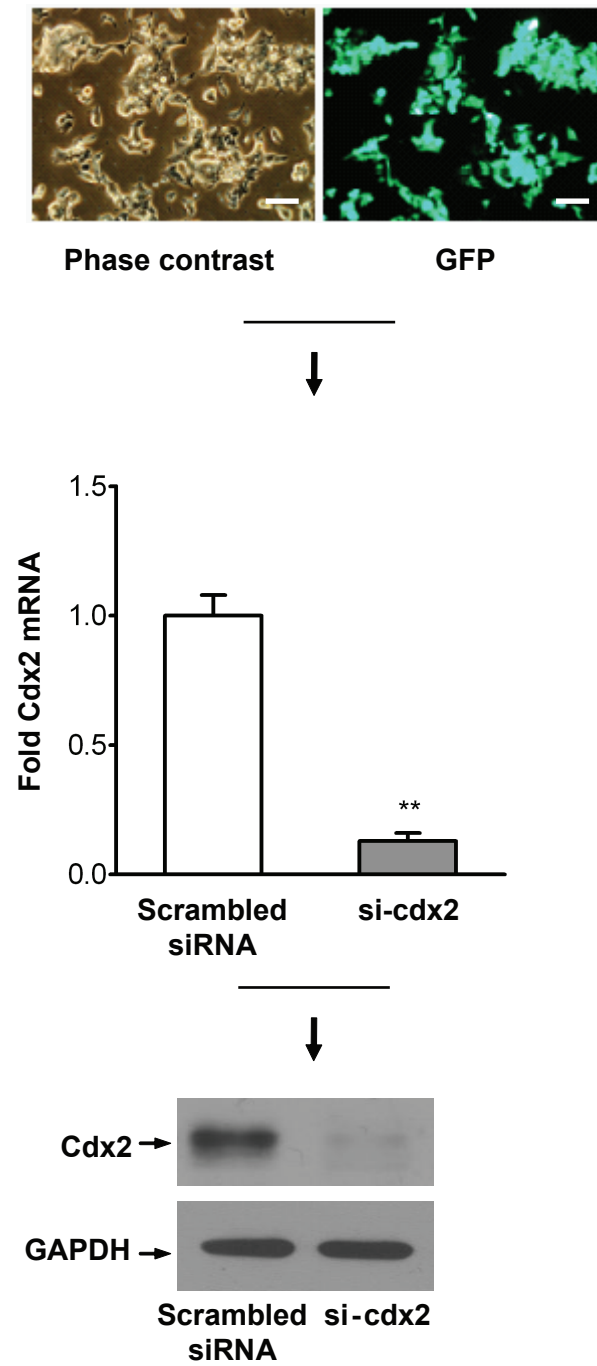

**Figure S2**

Supplement: Figure S2 — Cdx2 knockdown in LS174T colon cancer cells. (A) LS174T cells co-transfected with GFP expressing plasmid and scrambled siRNA or si-Cdx2 with images captured under appropriate filters (Phase contrast and GFP). The middle panel shows fold Cdx2 mRNA expression by RT-qPCR. β-actin was used as internal control. Gene expression changes were calculated using the comparative Ct method with β-actin as the reference gene and scrambled siRNA transfected cells as the calibrator. The bottom panel shows a western blot of Cdx2 from nuclear extract from the same cells. Histogram, mean ± SEM. Scale bar, 100 µm; ** P<0.02 (PDF) [file pone.0036075.s002.pdf]

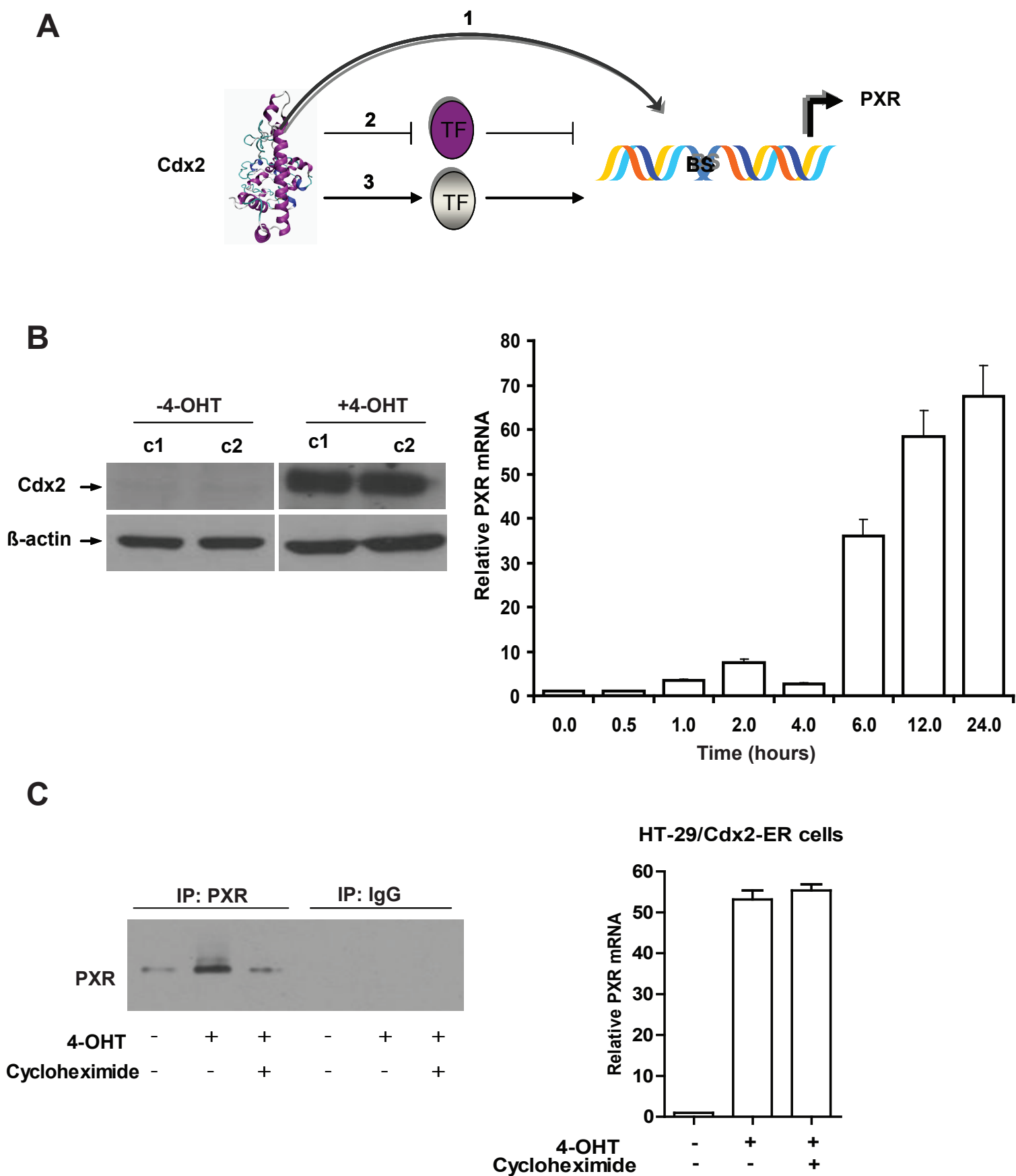

Figure S3

Supplement: Figure S3 — Cdx2 directly targets and induces the PXR promoter. (A) Schematic diagram illustrating possible mechanisms by which Cdx2 induces PXR mRNA, through its (1) direct action or (2, 3) indirect action(s) on the PXR promoter. BS: Cdx2 binding site on the PXR promoter. (B) Western blot of cell lysates from two clones (c1, c2) of HT-29/Cdx2-ER cells (obtained after sequential passage) with (+) or without (-) exposure to 4-OHT (hydroxytamoxifen) for Cdx2 and β-actin. The right panel shows time-dependent induction of PXR mRNA as assessed by RT-qPCR. (C) Immunoprecipitation blot of PXR from HT-29/Cdx2-ER cells exposed to cycloheximide and/or 4-OHT. Right panel shows fold expression of PXR mRNA by RT-qPCR. β-actin was used as internal or loading control for western blots. Gene expression changes were calculated using the comparative Ct method with β-actin as the reference gene and vehicle treated cells (or indicated as 0) as the calibrator. Histogram, mean ± SD. (PDF) [file pone.0036075.s003.pdf]

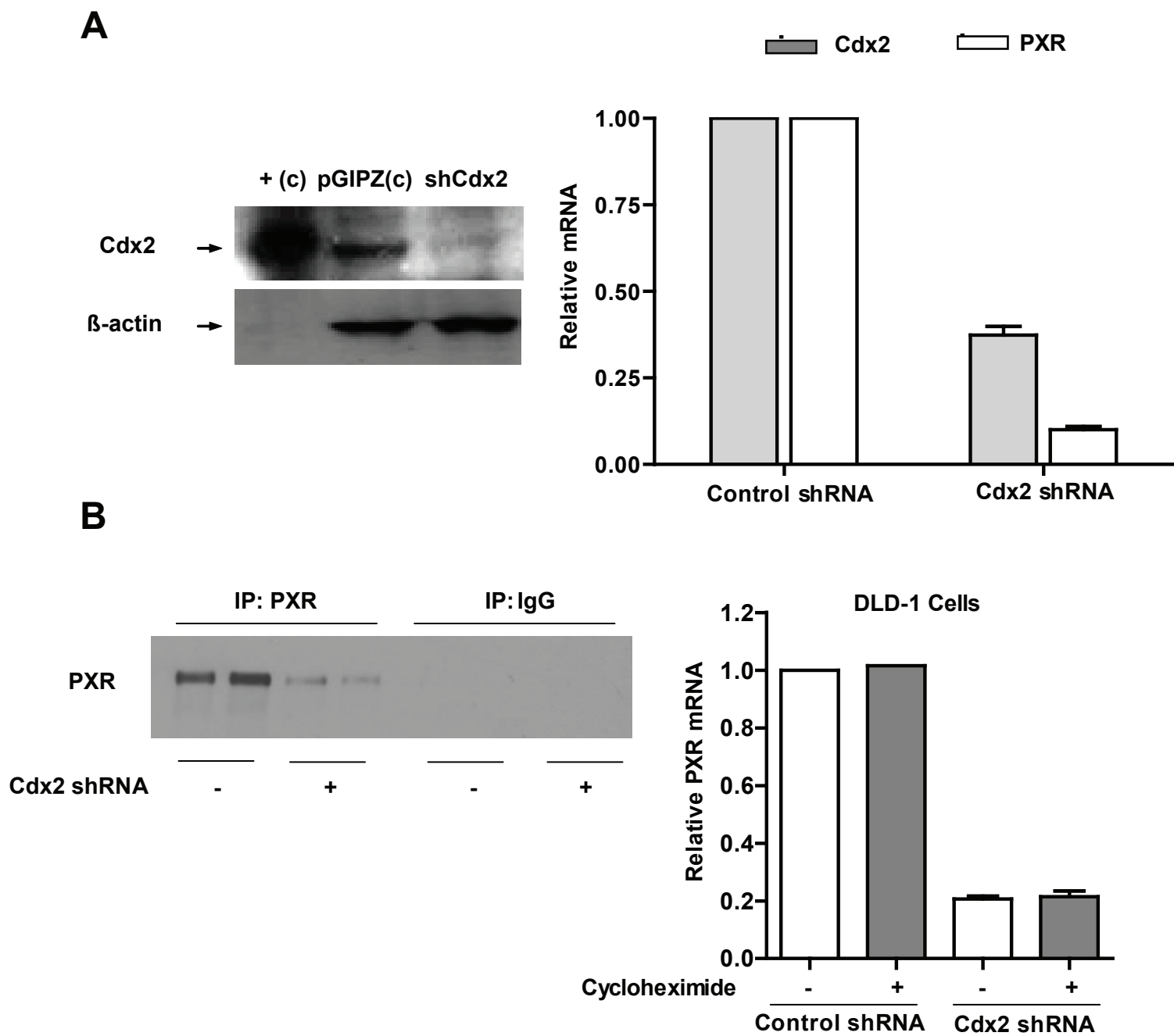

Figure S4

Supplement: Figure S4 — Cdx2 directly targets and induces the PXR promoter. (A) Left panel shows western blot of Cdx2 in cell lysates prepared from DLD-1 pGIPZ(c) (control cells) and shCdx2 infected cells. Right panel shows fold mRNA expression of Cdx2 and PXR by RT-qPCR from the same experiment. (B) Immunoprecipitation blot of PXR from control DLD-1 pGIPZ(c) and shCdx2 infected cells exposed to cycloheximide and/or 4-OHT. Right panel shows fold expression of PXR mRNA by RT-qPCR. β-actin was used as internal or loading control for western blots. Gene expression changes were calculated using the comparative Ct method with β-actin as the reference gene and vehicle treated cells (or indicated as 0) as the calibrator. Histogram, mean ± SD. (PDF) [file pone.0036075.s004.pdf]

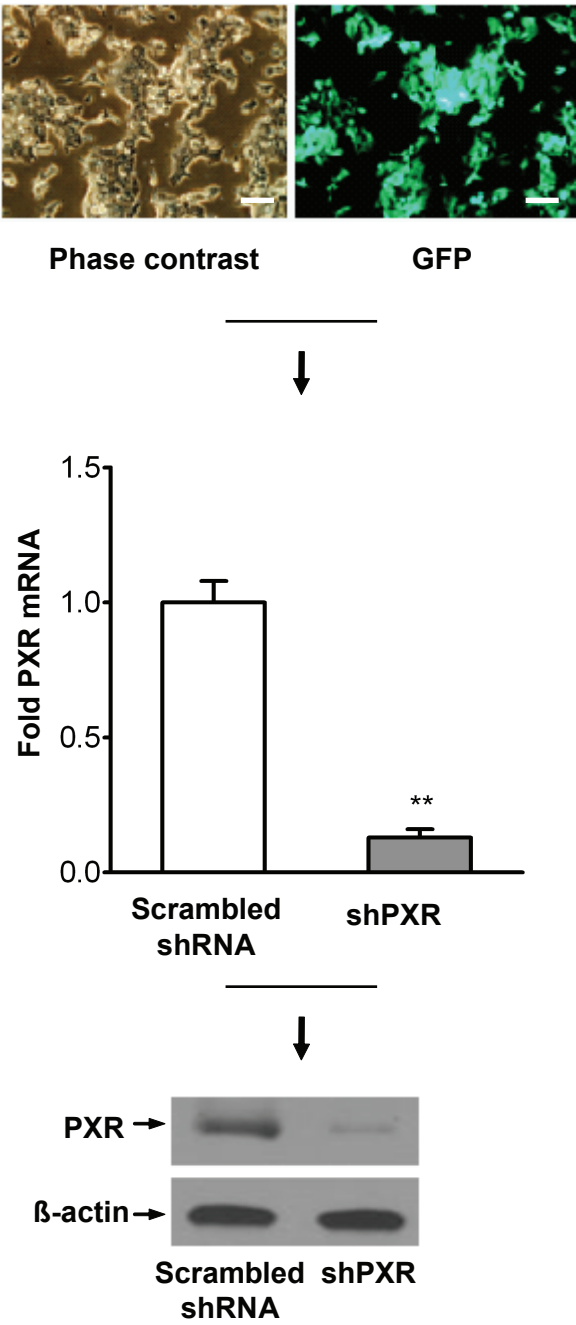

Figure S5

Supplement: Figure S5 — PXR knockdown in LS174T colon cancer cells. (A) LS174T cells co-transduced with GFP expressing plasmid and scrambled shRNA or shPXR with images captured under appropriate filters (Phase contrast and GFP). The middle panel shows fold PXR mRNA expression by RT-qPCR. β-actin was used as internal control. Gene expression changes were calculated using the comparative Ct method with β-actin as the reference gene and scrambled shRNA transfected cells as the calibrator. The bottom panel shows a western blot for PXR from nuclear extract from the same cells. Histogram, mean ± SEM. Scale bar, 100 µm; ** P<0.02 (PDF) [file pone.0036075.s005.pdf]

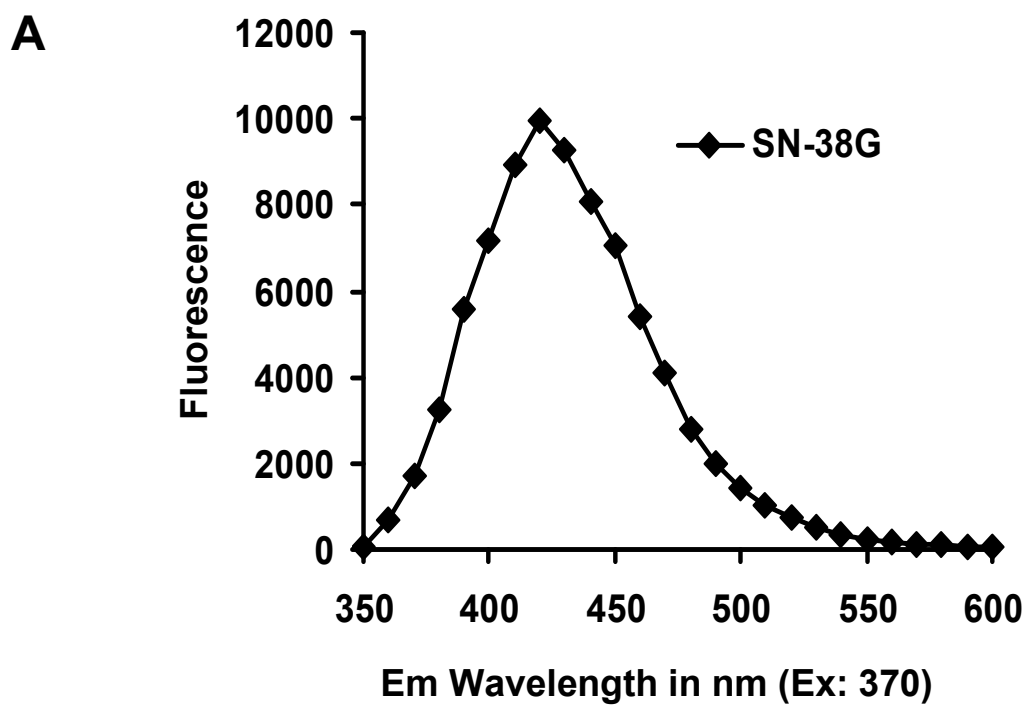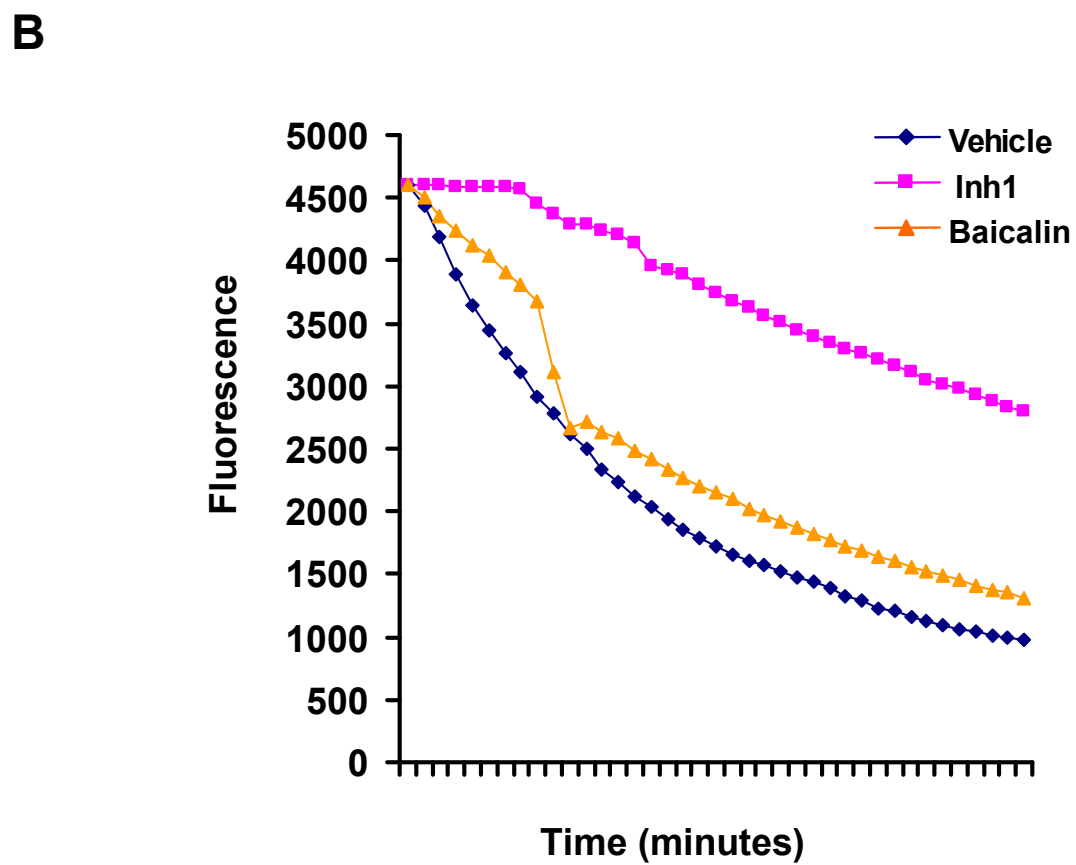

Figure S6

Supplement: Figure S6 — Baicalin does not decrease SN-38 glucuronide (SN-38G) emission spectra upon incubation with mouse feces. (A) SN-38G emission spectra in HEPES buffer. (B) Co-incubation of mouse feces with vehicle (HEPES buffer), Inh1 (10 µM), or Baicalin (10 µM). (PDF) [file pone.0036075.s006.pdf]

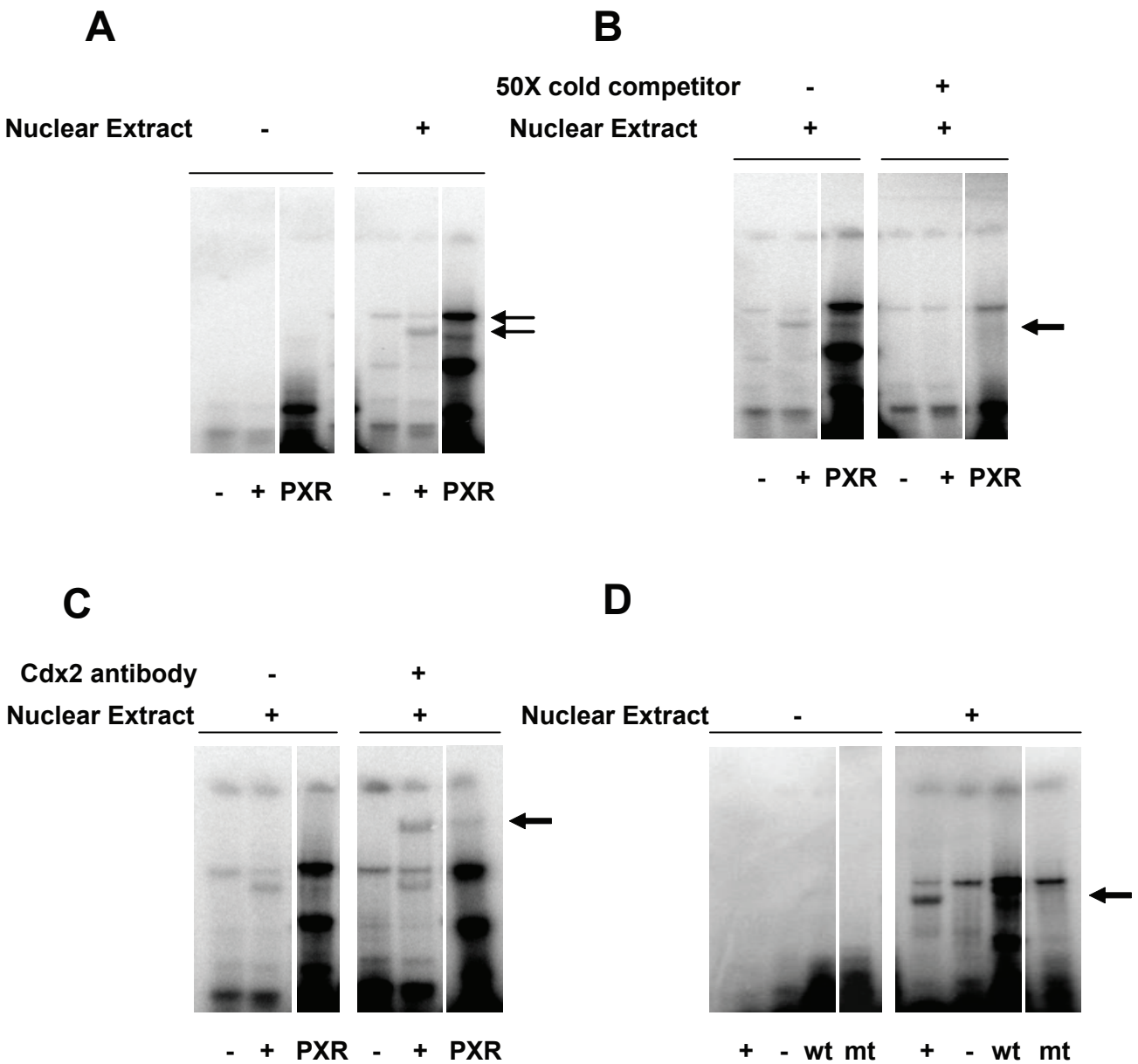

**Figure S7**

Supplement: Figure S7 — Cdx2 binds to binding Site1 (BS1) oligonucleotide probe by electrophoretic mobility shift assay (EMSA). (A) Black arrows demonstrate bands upon incubation of respective probes with and without LS174T colon cancer cell line nuclear extract. The lower arrow indicates band common to the positive control and PXR probe lane. (B) Black arrow (bold) demonstrates loss of band upon addition of 50-fold excess of cold PXR probe. (C) Black arrow (bold) demonstrates supershift band, identical to that observed in positive control lane, with the addition of Cdx2 antibody. (D) Black arrow (bold) demonstrates loss of band upon incubation of LS174T nuclear extracts with BS1 mutant oligo. -, negative control oligo; +, positive control oligo; wt, wild-type or PXR probe sequence from PXR promoter containing BS1; mt, PXR probe sequence mutant of BS1. (PDF) [file pone.0036075.s007.pdf]

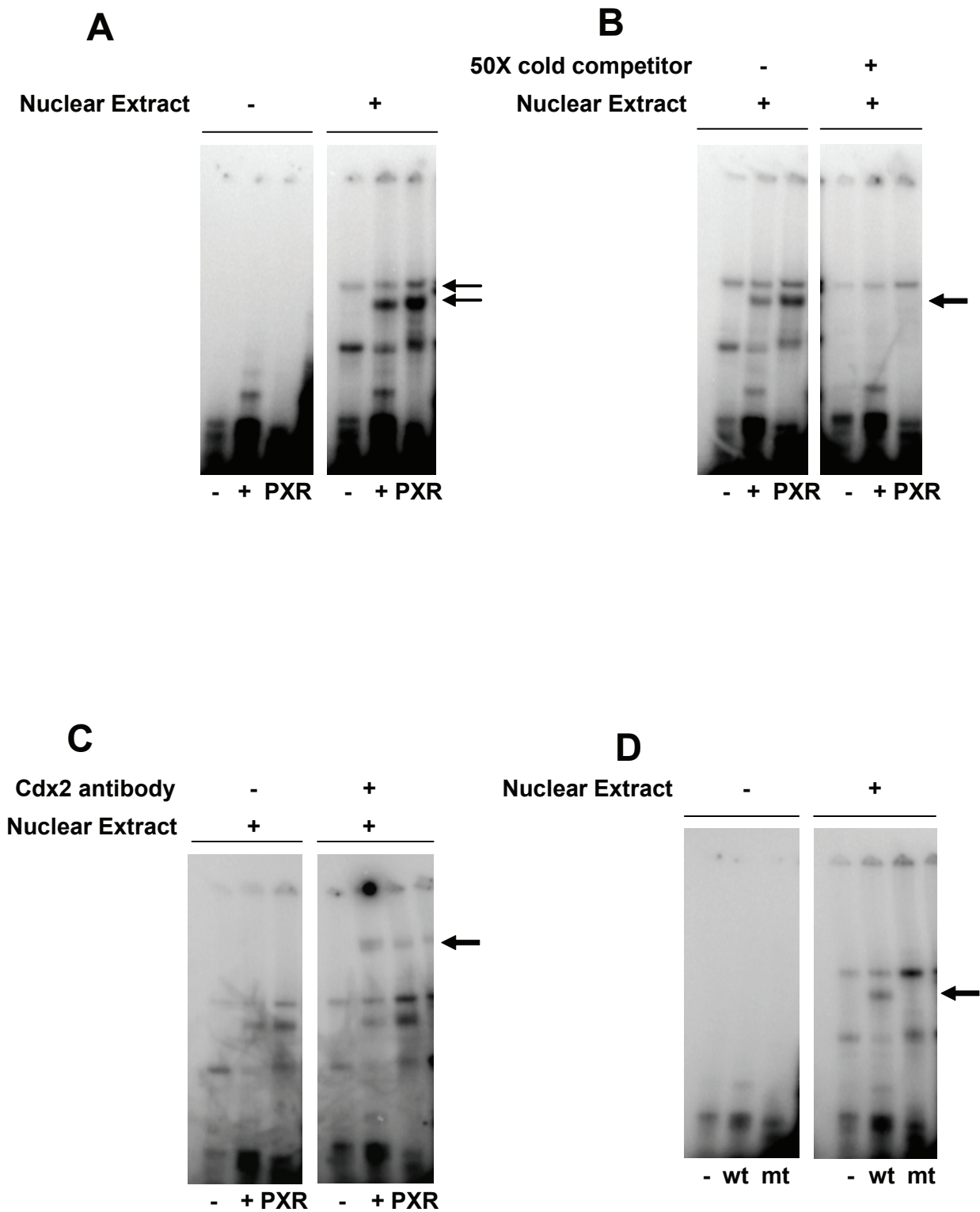

**Figure S8**

Supplement: Figure S8 — Cdx2 binds to binding Site2 (BS2) oligonucleotide probe by electrophoretic mobility shift assay (EMSA). (A) Black arrows demonstrate bands upon incubation of respective probes with and without LS174T colon cancer cell line nuclear extract. The lower arrow indicates band common to the positive control and PXR probe lane. (B) Black arrow (bold) demonstrates loss of band upon addition of 50-fold excess of cold PXR probe. (C) Black arrow (bold) demonstrates supershift band, identical to that observed in positive control lane, with the addition of anti-Cdx2 antibody. (D) Black arrow (bold) demonstrates loss of band upon incubation of LS174T nuclear extracts with BS2 mutant oligo. -, negative control oligo; +, positive control oligo; wt, wild-type or PXR probe sequence from PXR promoter containing BS2; mt, PXR probe sequence mutant of BS2. (PDF) [file pone.0036075.s008.pdf]
