# Supplementary material for: Alleviation of Gut Inflammation by Cdx2/Pxr Pathway in a Mouse Model of Chemical Colitis
Source: PLoS One. 2012 Jul 16;7(7):e36075. doi: 10.1371/journal.pone.0036075 (PMC3398007; doi:10.1371/journal.pone.0036075)
Supplement: Table S1 — Primer sequences for ChIP assay. (DOC) [file pone.0036075.s009.doc]

**Table S1. Primer sequences for ChIP assay.**

| Primer | Forward (5’ to 3’) | Reverse (5’ to 3’) | PCR amplicon size and conditions (Tm/cycle#) | ChIP detection | Cdx2 Binding Site (BS) |
| --- | --- | --- | --- | --- | --- |
| P1 | AAATGTGACTAGCAATTTTAAG- | GCTGTTGCTGTCAAGTTTCT | 290bp/55°C/40 | NO |  |
| P2 | CTCTTTGCTGTCAAAGGGTAA | TGGCTGCTCAGTTCAGTGTAGTA | 281bp/60℃/35 | NO |  |
| P3 | GGGAGAAGTTTCCTCCAAGA | GGCATAAGCCATTGTACCTG | 297bp/58℃/35 | NO |  |
| P4 | GACTCCCACCTACACCCTTCC | TCTCCTTCTTCATGCCGCTCT | 304bp/66℃/40 | NO |  |
| P5 | TGGGAAGTGCAAATTGGATAG | GACCACGATTGAGCAAACAGG | 232bp/58℃/35 | NO |  |
| P6 | CTGTTTGCTCAATCGTGGTC | AAAGCCGAATGTGGTGGATA | 195bp/58℃/35 | YES | BS2 |
| P7 | CCACTCCTGGTCAGCCTTCTGT | CCTTTGTTGAAACCACCTCCCT | 213bp/58℃/35 | NO |  |
| P8 | TCCCTTCAACAATACAGAACC | TGAGAAAATGCCCTGAGTTAT | 254bp/64℃/40 | NO |  |
| P9 | GGGATGGTAGTAACAGGACAA | AGCTAGAATCATGCCAGAGCA | 199bp/58℃/35 | NO |  |
| P10 | TCCCTCTTACCCTTATCTTTC | AACACTCTGTCCCTTCTATGC | 198bp/64℃/40 | NO |  |
| P11 | GCTGTTTCCTGTATGACCTC | GAGATAATTGGGTATTTGGT | 279bp/55℃/40 | YES | BS1 |
| P12 | GAAGAGGTATTGCGGTGTTA | TTGTAATGATGGCAAGTGTC | 206bp/55℃/40 | NO |  |
