# Supplementary material for: Alleviation of Gut Inflammation by Cdx2/Pxr Pathway in a Mouse Model of Chemical Colitis
Source: PLoS One. 2012 Jul 16;7(7):e36075. doi: 10.1371/journal.pone.0036075 (PMC3398007; doi:10.1371/journal.pone.0036075)
Supplement: Table S2 — Probe Sequences for EMSA. (DOC) [file pone.0036075.s010.doc]

**Table S2. Probe Sequences for EMSA.**

| **Binding Site** | **PXR Probe Sequence** | **Mutant PXR Probe Sequence** |
| --- | --- | --- |
| **BS1** | + strand: 5'- CCT TTA TCC CTC TCA AAC ATT ATA GTG ATT TAT GAA -3'  - strand: 5'- TTC ATA AAT CAC TAT AAT GTT TGA GAG GGA TAA AGG -3' | + strand: 5'- CCT TTA TCC CTC TCA AAC AT**G** **CGC** GTG ATT TAT GAA -3'  - strand: 5'- TTC ATA AAT CAC **GCG** **C**AT GTT TGA GAG GGA TAA AGG -3' |
| **BS2** | + strand: 5'- CAC CTG CCA TAA AGC ATC TTT AT -3'  - strand: 5'- ATA AAG ATG CTT TAT GGC AGG TG -3' | + strand: 5'- CAC CTG CCA **GC**A AGC ATC TTT AT -3'  - strand: 5'- ATA AAG ATG CTT **GC**T GGC AGG TG -3' |
