# Supplementary material for: Alleviation of Gut Inflammation by Cdx2/Pxr Pathway in a Mouse Model of Chemical Colitis
Source: PLoS One. 2012 Jul 16;7(7):e36075. doi: 10.1371/journal.pone.0036075 (PMC3398007; doi:10.1371/journal.pone.0036075)
Supplement: File S1 — File contains the following: Supporting Information Methods including primer sequences and shRNA sequences, Supporting Information Results and Supporting Information References. (DOC) [file pone.0036075.s011.doc]

**Alleviation of Gut Inflammation by Cdx2 /Pxr Pathway in a Mouse Model of Chemical Colitis**

Wei Dou*, Subhajit Mukherjee*, Hao Li, Madhukumar Venkatesh, Hongwei Wang, Sandhya Kortagere , Ariel Peleg, Sridhar S. Chilimuri, Zheng-Tao Wang, Ying Feng, Eric R. Fearon and Sridhar Mani

**Table of contents:**

Supporting Information Methods, including primer sequences and shRNA sequences

Supporting Information Results

Supporting Information References

Supporting Information Figures

*These authors contributed equally to this work.

**Supporting Information Methods**

**Cell lines And Reagents**

The cell lines were procured from American Type Culture Collection (ATCC, Rockville, MD) or as mentioned with the respective cell line. DLD-1 pGIPZ, shCdx2 infected cells and HT-29/Cdx2-ER cells were obtained and cultured according to the previously published methods [1]. Charcoal adsorbed fetal bovine serum (FBS), Baicalein (purity 99%), and DMSO were obtained from Sigma Aldrich; Baicalin (purity 99%) was provided by Shanghai University of Traditional Chinese Medicine; Dextran sulfate sodium (MW 36–50 KDa) was acquired from MP Biochemical LLC, Solon, OH; Dual-Luciferase reporter assay system, MultiTox-Fluor multiplex cytotoxicity assay system and Bright-Glo luciferase assay system were from Promega; Lipofectamine 2000, Platinum Taq DNA polymerase, SuperScript® III first-strand synthesis system and Trizol® reagent were obtained from Invitrogen; Protease inhibitor cocktail tablets were from Roche; nucleofector kit L was from Lonza, Cdx2 siRNA duplex (sc-43680) and Control siRNA duplex (sc-37007) were from Santa Cruz Biotechnology. For ChIP assay, human Cdx2 antibody (#3977) was from Cell Signaling and human RNA polymerase II antibody kit (GAH 111) was from SAbiosciences. Other antibodies used were as indicated: nonspecific rabbit IgG (sc-2027), β-actin (Abcam, ab8227), PXR (H-160, sc-25381). The microbial β-glucuronidase inhibitor, Inh1, was obtained from the Redinbo Laboratory (University of North Carolina, Chapel Hill, NC). ^32^p-ATP 250 μci (NEG502Z250) was obtained from Perkin Elmer (RNA polymerase II kit was purchased from SA Biosciences Corporation. 4-hydroxytamoxifen (4-OHT; Sigma) was a kind gift from Dr. Kalpana Ganjam of Albert Einstein College of Medicine, Bronx, NY. Cycloheximide (1 μg/mL) (Sigma) was used to assess whether PXR was a direct target of Cdx2 in HT-29/Cdx2-ER cells. All drugs were dissolved in 100% dimethyl sulfoxide (DMSO) and stored at -20°C. The final concentration of DMSO was < 0.2% in all experiments.

**In Silico Binding Site Analysis**

Five human NR1I2 sequence files generated by Gene2Promoter analysis [GXP_141828, GXP_141829, GXP_141830, GXP_489083 and GXP_2248527] were used to determine sites of CDX2 binding using Mat Inspector. Putative binding sites for transcription factor ($VCDXF) were identified using a stringent search setting, with matrix similarity set at > 0.75 and core similarity at >0.85 to minimize the identification of false positives. The matrix represents the DNA binding profile for $VCDXF, with the matrix similarity being the quality of a match between the matrix and the input sequence. Core similarity represents the quality of a match between the core sequence of a matrix (the four most conserved positions within a matrix) and the input sequence.

**Molecular Docking**

Three dimensional structure of hPXR co-crystalized with hyperforin was obtained from Protein data bank (PDB code: 1m13). The ligand hyperforin was removed and hydrogen atoms were added to prepare the structure for docking experiments. Further the hPXR structure was further refined via 1000 steps of conjugate gradient based energy minimization using Amber (version 9.0), with Amber charges as adopted in the Molecular Operating Environment (MOE) program (version 10; Chemical Computing Group, Montreal, Quebec, Canada). The binding site for hPXR has been well characterized based on structural information derived from the variety of ligands that it binds to. To adequately sample this promiscuous binding site 50 independent docking runs were performed for each ligand using the GOLD program (Genetic Optimisation for Ligand Docking) (version 4.1) [2]. The docked complexes were scored using Goldscore, Chemscore and a customizable scoring scheme that was previously designed to classify activators and non-activators of PXR [3,4,5,6,7,8,9]. The best ranking complexes were then energy minimized using Amber force field adopted in MOE program. In Figure 1, the Docking mode of Baicalein (shown in surface representation and colored atom type) and Baicalin (shown in licorice representation and colored atom type C – cyan, N – blue, O- red and H- white) in the binding site of hPXR (shown in ribbon representation and colored orange) is illustrated. Baicalein has hydrogen-bonded interactions with main chain atoms Val211, Leu209 and side chain of Tyr306 in addition to several favorable hydrophobic interactions such as Met243, Met246, Phe288, Leu206, Leu239 and Trp299 probably contributing to its high binding affinity. A schematic representation of the binding mode of Baicalin in PXR is shown. The covalent linkage of the O-glucuronide group induces a different conformation of Baicalin in comparison to Baicalein and hence leads to loss of favorable hydrogen bonding interactions, although some of the hydrophobic interactions are still maintained probably leading to a low activity at PXR.

**Gene Reporter Assays**

In brief, cells were transfected with 50 ng pGL4.19 PXR-luciferase reporter plasmids (-6.04kb, -3.81kb, -2.77kb, -0.72kb), 5pg pRL-TK internal control plasmid and for experiments using the -3.81kb reporter plasmid, increasing amounts of pCMV-Cdx2 plasmid (0, 10, 25, 50, 100, 150, 200ng). The deletion constructs were co-transfected with a fixed amount of pCMV-Cdx2 (100 ng). After 48 hours, the cells were harvested in passive lysis buffer (Promega) and luciferase activity was detected using the dual-luciferase reporter assay system using the Turner Bio-systems 20/20n Luminometer. Normalization and calculations of fold induction were performed as previously published [10]. For PXR transactivation in DPX2 cells, the latter were seeded into 96-well plate (white wall and clear bottom) at 3x10^4^/well, and exposed to Baicalein or Baicalin at concentrations of 1.1, 3.3, 10, 20, 30, 35, 40, 45, and 50 μM, respectively. After 48 hours of incubation, cells were rinsed in PBS, followed addition of 40 μl/well PBS and 10μl/well (5x) cell MultiTox-fluor reagent. The plate was then incubated for 37°C for 30 minutes and wells measured for live/dead cell florescence at 400ex/505em using Spectramax M5 Microplate Reader (MV05047, Molecular Devices). After completion of the assay, the same wells were exposed to Bright-Glo reagent (50μl/well) and cells lysed. Luciferase activity was determined by measuring luminescence using Spectramax M5 Microplate Reader (MV05047, Molecular Devices, Sunnyvale, CA). All gene reporter and viability assays were performed at least three separate times each in triplicate.

**Cdx2 siRNA Transfection**

The target sequence for Cdx2 specific siRNA duplex (Santa Cruz Biotechnology) comprised of a pool of 3 target-specific 19-25nt siRNAs. Non-targeting 20-25nt siRNA duplex (Santa Cruz Biotechnology) from a scrambled sequence was used as a negative control. 1x10^6^ LS174T cells were electroporated with 0.625μg (50 pmols) Cdx2 siRNA duplex or scramble control using Cell line nucleofector kit L on Nucleofector II (Amaxa Biosystems, Lonza Rockland, Inc. Rockland, ME). After 72 hour incubation, the efficacy of gene silencing was evaluated by real-time qPCR and Western blotting analysis as described below. To visually assess for transfection efficiency, separately, cells were also co-transfected with GFP-plasmid (CMV-GFP from Addgene, Cambridge, MA).

**RNA Preparation And Semi-quantitative RT-PCR (Semi- qPCR)**

LS174T cells were seeded in triplicate in six-well plates (density of 1.5 × 10^6^ cells/well). After an overnight incubation in charcoal adsorbed media, cells were exposed to baicalein or baicalin at concentrations of 1, 5, 10, 15, 25 and 50 μM, respectively, for 48 hours. Total RNA was extracted from cells using the RNeasy Mini kit (Qiagen, Valencia, CA) and/or TRIzol (Invitrogen, Carlsbad, CA), according to the manufacturer’s instructions. 3 µg of total RNA were reverse transcribed with random hexamer primers and SuperScript^TM^ III-RT (Invitrogen, Carlsbad, CA). Semi-qPCR was used to assess mRNA expressions of human Cdx2 with previously published primer sets [11]. The primers used for internal control, β-actins are: Forward: 5’-ctggggacgacatggagaaaa-3’ and Reverse: 5’-aaggaaggctggaagagtgc-3’. For human (h) cell lines, the following PCR conditions were used: 94˚C for 3 minutes, and then each cycle at 94˚C for 30 seconds, 56˚C for 30 seconds, 74˚C for 30 seconds, repeated for 25 cycles. The final cycle extension time (72˚C) was 5 minutes. All PCR products were resolved by 2% agarose gel electrophoresis and visualized using ethidium bromide (EtBr) staining.

**Real-Time quantitative (Q) PCR**

qPCR for human/mouse Cdx2, human/mouse PXR, human β-actin, mouse MDR1, mouse Cyp3a11, mouse TNFα, mouse IL-6, and mouse GAPDH, was performed using TaqMan reagents (Universal PCR master mix, FAM 5’ reporter flourochrome probe and tetramethylrhodamine [MGB] probe as the quencher flourochrome). Primers and minor groove binder/non-fluorescent quencher probes used for PCR amplification were obtained from Applied Biosystems using the following assays on demand: Hs01114265_gl (human PXR); Mm01344143_g1 (mouse PXR); Hs01078080_m1 (human Cdx2); Mm01212280_m1 (mouse Cdx2); Mm00443258_m1 (mouse TNFα); Mm01210733_m1 (mouse IL-6); (mouse MDR1); Mm00731567_m1 (mouse Cyp3a11); Mm99999913_g1 (mouse GAPDH); and Hs99999903_m1 (human β-actin) (Applied Biosystems, Carlsbad, California). Controls included RT-minus RNA samples. PCR reaction conditions for all assays were 50°C for 2 minutes, 95°C for 10 minutes, followed by 40 cycles of amplification (95˚C for 15 seconds then 60˚C for 1 minute). qPCR assays were in general performed at least two times with each well performed in triplicate.

**Western Blot Analysis**

Cell lysate was separated by SDS-PAGE and analyzed by immunoblotting as previously published [12,13,14]. Antibodies used in these studies were as follows: Cdx2 (#3977, Cell Signaling; 1:1000), PXR (H-160, sc-25381, Santa Cruz Biotechnology Inc.; 1:500), β-actin (ab8227, Abcam Inc.; 1:5000). β-actin was used to normalize the data for protein loading*.* After being incubated with horseradish peroxidase-conjugated secondary antibody, the proteins bound to the membrane were visualized using ECL method (GE Healthcare). Western blot analyses were performed at least three times each with at least three separate exposures. The intensity of the protein bands on the membrane was quantified using Image J software (NIH, <http://rsb.info.nih.gov/ij/>).

**Immunoprecipitation**

Cell lysates were prepared using RIPA buffer with Protease inhibitors. Proteins were separated by SDS-PAGE and analyzed by immunoblotting as previously published [12,13,14]. PXR H-160 antibody (sc-25381) was used for immunoprecipitation and PXR N-16 antibody (sc-9690) was used for western blot analysis. These antibodies were purchased from Santa Cruz Biotechnology (Santa Cruz, CA).

**Lenti-based shRNA knock-down System**

For PCR template sequences containing specific target sequences were obtained through the RNAi Codex website (codex.cshl.edu/scripts/newmain.pl), and the chosen sequences are as follows: PXR1.0: TGCTGTTGACAGTGAGCG**CGGCTACGCTGACAATCAGTTA**TAGTGAAGCCACAGATGTAT**AACTGATTGTCAGCGTAGCCT**TGCCTACTGCCTCGGA, (targets NM_4154….4175), scrambled: tgctgttgacagtgagcgcg**gtactgccgcaatgctata** atagtgaagccacagatgtat**tatagcattgcggcagtacc**ttgcctactgcctcgga, PXR2.0: TGCTGTTGACAGTGAGCG**CGGACCAGATCTCCCTGCTGAA**TAGTGAAGCCACAGATGTA**TTCAGCAGGGAGATCTGGTCCT**TGCCTACTGCCTCGGA, (targets [NM_003889](http://www.ncbi.nlm.nih.gov/nuccore?term=NM_003889) (2650...2671) scrambled: tgctgttgacagtgagcgcg**gcacaccggtcagcttact** atagtgaagccacagatgtat **agtaagctgaccggtgtgcc**ttgcctactgcctcgga. Bold letter sequences were derived from PXR target gene. PXR 1.0 and 2.0 were chosen after testing five from fifteen possible hairpin sequences. These sequences were cloned into Lenti: CMV–GFP plasmid individually or combined as previously published.[15]. The best results were obtained with PXR1.0/PXR2.0 combination cloned into a single lentivector (Figure S5). This system is specific to PXR as we have verified PXR protein re-expression using rescue plasmids as described previously [16].

**Lenti Virus Assembly and Transduction**

293T cells were cultured in Dulbecco’s Modified Eagle Medium (DMEM) supplemented with 10% fetal bovine serum (FBS). Lentivirus were generated through co-transfection of 293T cells with lentiviral plasmids (shRNA against PXR/scrambled PXR) and packaging plasmids (pMDLg/pRRE, pRSV-REV, and pMD2-VSVG or Trans-Lentiviral Packaging Mix- pTLA1-Pak, pTLA1-Enz, pTLA1-Env, pTLA1-Rev and pTLA1-TOFF, Open Biosystems) by standard calcium phosphate protocol [15]. After 48 hours of transfection, supernatant was collected and filtered (0.2 μm pore size). Virus titers were determined by infection of 293T cells with serial dilution of viral stock and the percentage of GFP positive cells were assessed by FACS. For transduction, LS174T cells were incubated with viral supernatants (shRNA PXR1/2 or scrambled shRNA) for 24 hours at 37°C in the presence of 8 μg/ml of polybrene (Sigma Aldrich). After 3 days, the percentage of GFP-positive cells was determined by fluorescence-activated cell sorting (FACS, BD Biosciences), as previously published (Figure S5) [15]. GFP positive cells were assessed and sorted by fluorescence-activated cell sorting (FACS, BD Biosciences). GFP-positive cells were cultured and knockdown efficacy was assessed by real-time qPCR and immunoblot.

**Chromatin Immunoprecipitation (ChIP) Assay**

Briefly, in the fast ChIP method, the cells are first cross-linked with formaldehyde and then lysed. Fractions containing nuclear pellets were isolated, and the chromatin is sheared. The chromatin samples were incubated with antibodies in an ultrasonic bath followed by centrifugation to obtain pre-cleared samples. These samples were mixed with protein ‘A’ beads. After several washes, Chelex 100 suspension is added to the beads. The suspension is boiled and then allowed to cool. After shaking and repeated boiling, the centrifuged samples are analyzed for PCR-ready DNA. We initially used a 35-cycle PCR to detect interaction but observed nonspecific interactions (i.e., IgG plus rifampicin-treated samples), so we reduced the cycle number to 30. For semi-quantitative PCR, several PCR amplicon sizes of 189-304 bp (putative Cdx2 binding site on the proximal PXR promoter) were found. The input reflects 0.5% of the total lysate after sonication. IP controls included H_2_O (water) and nonspecific IgG (same class as the PXR antibody). Details of the PCR conditions and primer sequences are described in Table S1. The negative control in the ChIP assay was the PXR intron 1 amplicon (293 bp) defined by primers (these primer sets are unable to amplify RNA, data not shown): Forward: 5’-GAGGTGTCACTGCCATCTTCAT-3’;

Reverse: 5’-GAGGCTGCTTACTCTGGGTTTT-3’

The positive control in this assay was Cdx2 binding site within the Furin promoter (188 bp amplicon): Forward: 5’-CTCTTCACGCCATTCCTTTC-3’; Reverse: 5’-TGCTTGAAGGGCATTATTTTG-3’

**RNA Pol II Recruitment Assay**

Following Cdx2 (and scrambled) siRNA transfection, LS174T cells were expanded and ChIP assays were performed as described above. Equal aliquots of soluble chromatin was subjected to overnight immunoprecipitation with either anti-Pol II antibody body (GAH 111, SAbiosciences) or non-specific IgG. DNA was purified and semi-quantitative PCR was performed to detect two putative Pol II binding sites on PXR promoter [17] . The corresponding primers (TATA1 with 213bp amplicon and TATA2 with 241bp amplicon) were used to amplify both Pol II (TATA) binding sites on PXR promoter:

TATA1-Forward: 5’-CCACTCCTGGTCAGCCTTCTGT,

TATA1-Reverse: 5’-CCTTTGTTGAAACCACCTCCCT;

TATA2-Forward: 5’- TGCTGGACTTGGGACTTAGGAG,

TATA2-Reverse: 5’- CTGCCTTGTTCAGGACAGGAGTA.

The PCR conditions included a hot start (94˚C for 3 minutes) followed by 35 cycles of 94˚C for 30 seconds, 58˚C for 20 seconds, 72˚C for 30 seconds, then 72˚C for 3 minutes.

**Electrophoretic Mobility Shift Assay (EMSA)**

Double-stranded oligonucleotides of specific response elements within PXR were used as probes (Table S2); positive and negative control oligos of human vitamin D receptor sucrase-isomaltase footprint 1 (hVD-SIF-1) were synthesized as described previously [18]. The oligonucleotides were synthesized by Invitrogen. These oligonucleotide fragments were labeled with [γ-^32^P] ATP (PerkinElmer, Massachusetts, USA) using T4 polynucleotide kinase (New England Bio labs, Woburn, MA). LS174T nuclear extract (5 μg) was incubated with the radiolabeled probe in binding buffer (10 mM Tris-Cl, pH 8.0, 150 mM KCl, 0.2 mM dithiothreitol, 0.5 mM EDTA, 14% glycerol) in a final volume of 10 µl for 30 minutes at room temperature. In some experiments, a 50-fold excess of cold probe was used, or a supershift of the index (Cdx2 binding) band was determined using a Cdx2 antibody. The reaction mixture was then subjected to electrophoresis on a 4-20% gradient native gel (Bio-Rad, Hercules, CA). The gel was dried and analyzed by a phosphoimager (STORM 860, Molecular Dynamics).

The negative control probe sequences are:

- control hVD-SIFM

hVD-SIFM+:

5'- GGT CAC AAG CAA AAC TGC TTT CTT A -3'

hVD-SIFM-:

5'- TAA GAA AGC AGT TTT GCT TGT GAC C -3'

The positive control probe sequences are:

+ control hVD-SIF1 (human vitamin D receptor sucrase-isomaltase footprint 1)

hVD-SIF+:

5'- GGT CAC AAT AAA AAC TTA TTT CTT A -3'

hVD-SIF-:

5'- TAA GAA ATA AGT TTT TAT TGT GAC C -3'

**PXR Promoter Deletion Constructs**

All fragments were PCR amplified from PXR BAC clone (*NR1I2*, Clone ID: RP11-169N13, bacpac.chori.org) using different forward primers with *kpn* I restriction sites (-6.04 kb forward primer: 5'- AAG GTA CCC TTG AAA ACA GTG AGA GAG AGG -3'; -3.81 kb forward primer: 5′- GATCGGTACCCACTGAACTGAGCAGCCACCAAATGCAAT-3′; -2.77 kb forward primer: 5′-GATCGGTACCGGCAAAGTATAGAAATGGAGGTCAAGGCCTCA-3′; and -0.72 kb forward primer: 5′-GATCGGTACCCTGGACTTGGGACTTAGGAGGGGCAATGGA-3′ and a single reverse primer with *Hind* III restriction site (5′-GATCAAGCTTGGCCTCAGGGGGCTGCCAGCACTAACCCAG-3′). PCR fragments were subsequently enzyme digested with *Kpn* I and *Hind* III (NEB, Ipswich, MA) and cloned into the pGL4.19 plasmid (Promega, Madison, WI), upstream of the luciferase reporter gene. All generated constructs were verified by DNA sequencing. PCR conditions used for all deletion fragment generations were similar and are as follows: initial denaturation at 94°C for 3 minutes followed by 35 cycles consisting of denaturation at 94°C for 45 s, primer annealing at 55°C for 45 s, elongation at 72°C for 3 minutes and final elongation at 72°C for 10 minutes.

**Site-Directed Mutagenesis**

Cdx2 binding sites on the -6.04-kb deletion construct of pGL-4.19 PXR promoter were mutated using the QuickChangeII XL Mutagenesis Kit from Agilent Technologies (Santa Clara, CA) as instructed. Primers for Cdx2 binding site 1 (BS1) are: 5'- CCC TCT CAA ACA T**GC** **GC**G TGA TTT ATG AAA ATA TAT G -3', 5'- CAT ATA TTT TCA TAA ATC AC**G** **CGC** ATG TTT GAG AGG G -3'. Primers for Cdx2 binding site 2 (BS2) are: 5'- CCC TCA CCT GCC A**GC** AAG CAT CTT TAT AAA C -3', 5'- GTT TAT AAA GAT GCT T**GC** TGG CAG GTG AGG G -3'.

**β-Glucuronidase Enzyme Assay**

Feces from mice treated with distilled water, baicalein, baicalin (20 mg/kg/day for 5 days by oral gavage) or inhibitor 1 (Inh1) (10 µg twice a day) were collected on day 5, after the mice were euthanized. The fecal pellets (collected from total colon) were suspended overnight at 4°C in HEPES buffer (pH 7.5). The suspension was centrifuged, and the cleared supernatant was collected and stored at 4°C for immediate use. The total protein content of the supernatant was determined using the BioRad protein reagent assay (Bio-Rad, Hercules, CA). The microbial β-glucuronidase assay was performed using SN-38 glucuronide (SN-38G) as the substrate (1.0 μM). SN-38G (2.0 μM) has an emission maximum at 418 nm when excited at 370 nm (Figure S6A). Twenty-five micrograms of fecal supernatant, stored at 4°C was mixed with 1.0 μM SN-38G in a total volume of 1 ml (200 mM HEPES buffer), and fluorescence intensity was measured against the incubation time (upto 1 h 35 minutes) of SN-38G (Spectra Max M5, Molecular Devices Inc., Sunnyvale, CA).

**Immunohistochemistry**

Hematoxylin and Eosin (H&E) staining was carried out in the histopathology core facility of Albert Einstein College of Medicine, Bronx, NY as described previously [16].

**Supporting Information Results**

**Baicalein, in contrast to baicalin, induces PXR mRNA expression through Cdx2**

The HT-29/Cdx2-ER cell line was incubated with 4- hydroxytamoxifen (4-OHT), which resulted in a marked induction of Cdx2 protein expression (Figure S3B). PXR mRNA induction was observed within 12 hours of 4-OHT treatment and increased until day 2 (Figure S3B). Cycloheximide did block the induction of PXR protein expression in 4-OHT–treated HT-29/Cdx2-ER cells; however, blockade of new protein synthesis by cycloheximide treatment did not inhibit the induction of PXR transcripts (Figure S3C). These observations are consistent with the notion that PXR is a direct or primary target gene regulated by Cdx2. As a corollary, DLD-1 derived lines with constitutively expressing Cdx2 shRNA were used to confirm reciprocal effects of Cdx2 and PXR. The DLD-1/Cdx2 shRNA cell line expresses negligible amounts of Cdx2 protein and mRNA when compared with the parental control cell line DLD-1/pGIPZ (Figure S4A, left panel). There is strong repression of PXR mRNA (Figure S4A, right panel) and protein (Figure S4B, left panel) expression in the Cdx2 shRNA cells as compared with control cells. The blockade of new protein synthesis by cycloheximide treatment did not affect PXR transcript or protein abundance in the DLD-1/Cdx2 shRNA cell line (Figure S4B).

**Cdx2 binds to specific PXR proximal promoter element**

The initial EMSA shows three bands (Figures S7A and 8A, respectively). Note, the positive control band exclusively identifies two bands corresponding to the top bands for BS1 (Figure S7A) and BS2 (Figure S8A), thereby excluding the third band as being specific to Cdx2. Cold competitor probe essentially quenches signals for the lower two bands (Figures S7B and S8B). The supershift assay using the Cdx2 antibody clearly revealed a shift in the middle band (denoted by the single black arrow), which is identical to the shift observed in the positive control lane (Figures S7C and S8C). To further validate this finding, we mutated key residues on the oligonucleotide that predict Cdx2 binding, and again, we observe a loss of signal for the middle band (Figures S7D and S8D). Since the middle band correlates with the index band for the positive control, we conclude that it is the probe sequence that binds specifically to Cdx2.

**Supporting Information References**

1. Takakura Y, Hinoi T, Oue N, Sasada T, Kawaguchi Y, et al. (2010) CDX2 regulates multidrug resistance 1 gene expression in malignant intestinal epithelium. Cancer Res 70: 6767-6778.

2. Jones G, Willett P, Glen RC, Leach AR, Taylor R (1997) Development and validation of a genetic algorithm for flexible docking. J Mol Biol 267: 727-748.

3. Chekmarev D, Kholodovych V, Kortagere S, Welsh WJ, Ekins S (2009) Predicting inhibitors of acetylcholinesterase by regression and classification machine learning approaches with combinations of molecular descriptors. Pharm Res 26: 2216-2224.

4. Ekins S, Kortagere S, Iyer M, Reschly EJ, Lill MA, et al. (2009) Challenges predicting ligand-receptor interactions of promiscuous proteins: the nuclear receptor PXR. PLoS Comput Biol 5: e1000594.

5. Kortagere S, Chekmarev D, Welsh WJ, Ekins S (2009) Hybrid scoring and classification approaches to predict human pregnane X receptor activators. Pharm Res 26: 1001-1011.

6. Kortagere S, Krasowski MD, Ekins S (2009) The importance of discerning shape in molecular pharmacology. Trends Pharmacol Sci 30: 138-147.

7. Peng Y, Zhang Q, Arora S, Keenan SM, Kortagere S, et al. (2009) Novel delta opioid receptor agonists exhibit differential stimulation of signaling pathways. Bioorg Med Chem 17: 6442-6450.

8. Plourde NM, Kortagere S, Welsh W, Moghe PV (2009) Structure-activity relations of nanolipoblockers with the atherogenic domain of human macrophage scavenger receptor A. Biomacromolecules 10: 1381-1391.

9. Westrich L, Gil-Mast S, Kortagere S, Kuzhikandathil EV (2010) Development of tolerance in D3 dopamine receptor signaling is accompanied by distinct changes in receptor conformation. Biochem Pharmacol 79: 897-907.

10. Venkatesh M, Wang H, Cayer J, Leroux M, Salvail D, et al. In vivo and in vitro characterization of a first-in-class novel azole analog that targets pregnane X receptor activation. Mol Pharmacol 80: 124-135.

11. Wong NACS, Wilding J, Bartlett S, Liu Y, Warren BF, et al. (2005) CDX1 is an important molecular mediator of Barrett's metaplasia. Proceedings of the National Academy of Sciences of the United States of America 102: 7565-7570.

12. Huang H, Wang H, Sinz M, Zoeckler M, Staudinger J, et al. (2007) Inhibition of drug metabolism by blocking the activation of nuclear receptors by ketoconazole. Oncogene 26: 258-268.

13. Wang H, Li H, Moore LB, Johnson MD, Maglich JM, et al. (2008) The phytoestrogen coumestrol is a naturally occurring antagonist of the human pregnane X receptor. Mol Endocrinol 22: 838-857.

14. Wang H, Huang H, Li H, Teotico DG, Sinz M, et al. (2007) Activated pregnenolone X-receptor is a target for ketoconazole and its analogs. Clin Cancer Res 13: 2488-2495.

15. Sun D, Melegari M, Sridhar S, Rogler CE, Zhu L (2006) Multi-miRNA hairpin method that improves gene knockdown efficiency and provides linked multi-gene knockdown. Biotechniques 41: 59-63.

16. Wang H, Venkatesh M, Li H, Goetz R, Mukherjee S, et al. Pregnane X receptor activation induces FGF19-dependent tumor aggressiveness in humans and mice. J Clin Invest 121: 3220-3232.

17. Kurose K, Koyano S, Ikeda S, Tohkin M, Hasegawa R, et al. (2005) 5' diversity of human hepatic PXR (NR1I2) transcripts and identification of the major transcription initiation site. Mol Cell Biochem 273: 79-85.

18. Yamamoto H, Miyamoto K, Li B, Taketani Y, Kitano M, et al. (1999) The caudal-related homeodomain protein Cdx-2 regulates vitamin D receptor gene expression in the small intestine. J Bone Miner Res 14: 240-247.
